# ISCU-p53 axis orchestrates macrophage polarization to dictate immunotherapy response in esophageal squamous cell carcinoma

**DOI:** 10.1038/s41419-025-07787-7

**Published:** 2025-06-20

**Authors:** Jialiang Luo, Xu Zhang, Zhicheng Liang, WeiTao Zhuang, Mingxin Jiang, Min Ma, Shuying Peng, Shujie Huang, Guibin Qiao, Qingyun Chen

**Affiliations:** 1 Institute of Medical Research, Guangdong Provincial People’s Hospital (Guangdong Academy of Medical Sciences), Southern Medical University, Guangzhou, Guangdong China; 2 Department of Medical Laboratory, School of Laboratory Medicine and Biotechnology, Southern Medical University, Guangzhou, Guangdong China; 3 Department of Thoracic Surgery, State Key Laboratory of Oncology in South China, Sun Yat-sen University Cancer Center, Guangzhou, Guangdong China; 4 Department of Immunology, School of Basic Medical Sciences, Southern Medical University, Guangzhou, Guangdong China; 5 Department of Medical Oncology, State Key Laboratory of Oncology in South China, Guangdong Provincial Clinical Research Center for Cancer, Sun Yat-sen University Cancer Center, Guangzhou, Guangdong China; 6 School of Medicine, South China University of Technology, Guangzhou, Guangdong China; 7 Department of Thoracic Surgery, Guangdong Provincial People’s Hospital (Guangdong Academy of Medical Sciences), Southern Medical University, Guangzhou, Guangdong China; 8 Department of Thoracic Surgery, Zhujiang Hospital, Southern Medical University, Guangzhou, Guangdong China

**Keywords:** Oncogenesis, Tumour immunology

## Abstract

Immunological heterogeneity in esophageal squamous cell carcinoma (ESCC) poses a significant challenge to the efficacy and response to immunotherapy. In this study, we used single-cell RNA sequencing to uncover substantial heterogeneity in the tumor microenvironments (TMEs) among patients received PD-1 inhibitor with partial response (PR), stable disease (SD), and that who underwent surgery without prior therapy. Notably, tumors classified as SD demonstrated an immunosuppressive environment, characterized by a higher prevalence of M2-like macrophages and lower frequencies of T and B cells, especially PD1^high^CD8^+^ T cells. These PD1^high^CD8^+^ T cells were found to frequently engage with macrophages within the TMEs. Focusing on macrophages, we observed elevated expression of the Iron-Sulfur Cluster Assembly Enzyme (ISCU) in macrophages infiltrating SD tumors. ISCU was identified as a promoter of M2 macrophage polarization in a p53-dependent manner. Mechanistically, ISCU sequestrates p53 in the cytoplasm, reducing its nuclear location and relieving transcriptional repression of xCT and Arg1. Consequently, the increased expression of xCT and Arg1 modulates macrophage sensitivity to ferroptosis and the arginine metabolic pathway, thus affecting macrophage differentiation and inflammatory responses. Furthermore, inhibition of ISCU expression was found to repolarize macrophages, enhance CD8^+^ T cell cytotoxicity, and boost the efficacy of anti-PD-1 antibody. Collectively, our findings highlight the complex interplay within ESCC TMEs and suggest that targeting ISCU might be a novel strategy to reprogram the immunosuppressive TME, potentially improving immunotherapy outcomes in ESCC patients.

**Figure:**
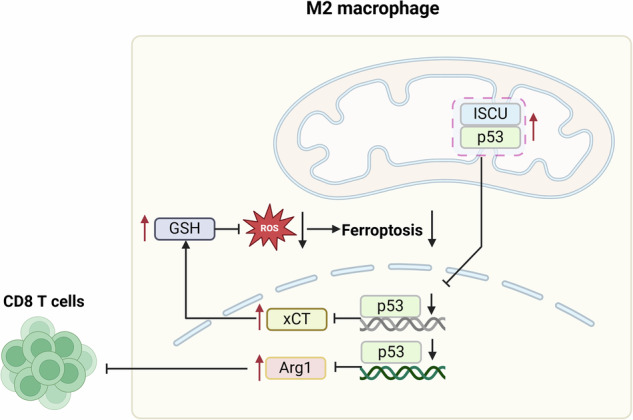
Schematic illustration of the mechanism by which ISCU facilitates M2 macrophage polarization. ISCU interacted with p53, promoting its retention in the cytoplasm during M2 macrophage polarization. This nuclear reduction of p53 results in the upregulation of xCT and Arg1, as both are negatively regulated at the transcriptional level by p53. The increased expression of xCT and Arg1 modulates macrophage sensitivity to ferroptosis and the arginine metabolic pathway, respectively, thus affecting macrophage differentiation and inflammatory responses. The graphical abstract was created with BioRender.com.

Schematic illustration of the mechanism by which ISCU facilitates M2 macrophage polarization. ISCU interacted with p53, promoting its retention in the cytoplasm during M2 macrophage polarization. This nuclear reduction of p53 results in the upregulation of xCT and Arg1, as both are negatively regulated at the transcriptional level by p53. The increased expression of xCT and Arg1 modulates macrophage sensitivity to ferroptosis and the arginine metabolic pathway, respectively, thus affecting macrophage differentiation and inflammatory responses. The graphical abstract was created with BioRender.com.

## Introduction

Esophageal squamous cell carcinoma (ESCC) is a prevalent and highly lethal malignant disease. Neoadjuvant chemo-radiotherapy combined with surgery has become the standard treatment for locally advanced ESCC patients [1]. Nevertheless, this treatment approach may enhance toxicity levels and result in severe side effects [2]. Immunotherapy has shown promising results in the treatment of advanced esophageal cancer [3]. The use of immune checkpoint inhibitor combined with chemotherapy as neoadjuvant therapy in advanced ESCC has showed significantly increased rates of pathological complete response (pCR) and major pathological response (mPR) compared to routine neoadjuvant therapy. However, different trials showed that ESCC patients have different amounts of benefit from immune checkpoint inhibition [4]. There is still a lack of research on the immunological heterogeneity and complexity in ESCC in response to immunotherapy.

Notably, exhausted CD8^+^ T cells are the primary target cells in immunotherapy. In the case of chronic infection or cancer, CD8^+^ T cells are constantly exposed to antigen and inflammatory signals, which often leads to T cell exhaustion. An important characteristic of exhausted CD8^+^ T cells is the simultaneous expression of multiple checkpoint molecules, which protect T cells from uncontrolled immune activation. This feature can also be used by tumors, making them resistant to T cell attack [5, 6]. Apart from prolonged antigen stimulation, the dysfunction of CD8^+^ T cells induced by tumors is also influenced by the immunosuppressive factors in tumor microenvironments (TMEs), such as myeloid-derived suppressor cells (MDSCs), tumor-associated macrophages (TAMs), regulatory T cells, IL-10, TGF-β and indoleamine 2,3-dioxygenase [7]. Among these components, macrophages are the most abundant innate immune cells in the TMEs. TAMs of the M2 phenotype play a dual role as “tumor promoters” and “immune suppressors”. This dual role arises from their ability to initiate tumor growth and regulate the TMEs by expressing various cell surface receptors, secreted cytokines, chemokines, and enzymes that control the recruitment and function of multiple immune cell subtypes [8]. Numerous studies have demonstrated that TAMs pose a significant obstacle to maximizing the clinical potential of immunotherapy [9, 10]. Therefore, gaining a deeper understanding of the heterogeneity of macrophages in TMEs following immunotherapy could provide valuable insight for improving the effectiveness of immunotherapy.

ISCU is known to play a crucial role in the assembly and maintenance of iron-sulfur clusters, which are essential cofactors for a wide range of proteins involved in various biological processes [11, 12]. Previous research has shown that ISCU expression is repressed by miR-210, leading to the inhibition of mitochondrial respiration and related downstream functions [11, 13]. In macrophages, ISCU is involved in orchestrating OXPHOS and modulating inflammatory responses to infectious diseases. Although the importance of ISCU in mitochondrial respiration, energy production, and inflammatory response is recognized [11], direct knowledge of its effects on macrophage polarization and function remains limited, and its underlying mechanisms are not well characterized.

Ferroptosis is designated as a regulated cell death characterized by lipid ROS overload, mitochondrial shrinkage, and decreased anti-oxidant markers (GPX4 and SLC7A11) [14]. Unlike apoptosis, ferroptosis involves the extensive release of oxidized lipid mediators and inflammatory cytokines [15]. Recent research has elucidated the role of p53 in regulating ferroptosis. Mechanistically, p53 transcriptionally represses SLC7A11, a key component of the cystine/glutamate antiporter system Xc−. This repression reduces cystine uptake, subsequently lowering GSH levels and increasing lipid peroxidation, thereby promoting ferroptosis [16]. p53 also influences metabolic pathways impacting ferroptosis, such as regulating the expression of ferredoxin reductase (FDXR), a p53 target gene that maintains mitochondrial iron homeostasis and promotes ferroptosis [17]. Although the proinflammatory role of ferroptosis in macrophages has been well established [15, 18], the regulatory relationships between ISCU and ferroptosis, as well as their roles in modulating the macrophage anti-tumor response, remain unclear.

In this study, we analyzed immunological heterogeneity in ESCC tumor formation after neoadjuvant therapy, and identified a new physiological function of ISCU in positively regulating M2 macrophage polarization. Mechanistically, ISCU interacts with p53, reducing its nuclear translocation, which results in the upregulation of xCT and Arg1, as both are negatively regulated at the transcriptional level by p53. The increased expression of xCT and Arg1 modulates macrophage sensitivity to ferroptosis and the arginine metabolic pathway, respectively. These enhance the anti-inflammatory phenotype, subsequently dampening CD8^+^ T cell activation and the response to immunotherapy.

## Results

### ScRNA-seq of single cells isolated from ESCC

**Fig. 1:**
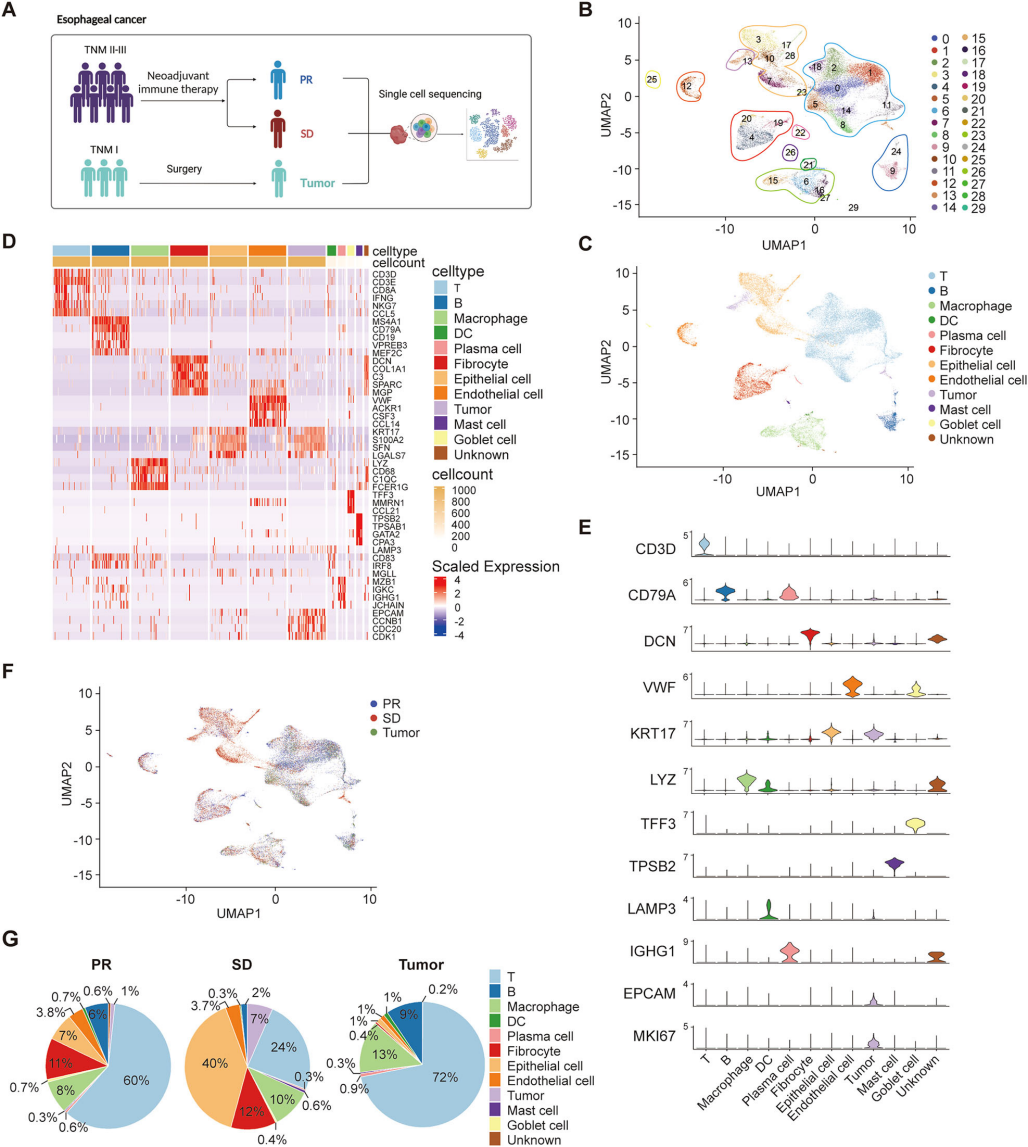
Overview of the ESCC ecosystem characterized by scRNA-seq. **A** Schematic representation of the overall study design. UMAP plots of 38,342 cells, colored by cell sub-cluster (**B**) and main cell type (**C**). **D** Heatmap displaying the expression of marker genes in main cell types. **E** Violin plots showing the expression of marker genes in identified main cell types. **F** UMAP plot of 38,342 cells, colored by sample origin. **G** Pie chart showing the proportion of each main cell type within the sample source.

We performed scRNA-seq on cells from four ESCC patients who received neoadjuvant camrelizumab combined with chemotherapy (two with partial response [PR] and two with stable disease [SD]) and one patient without neoadjuvant therapy (surgical patient) (Fig. 1A ideogram). The clinical information was shown in Supplementary Table 1. Using 10x Genomics sequencing, we retained 38,342 high-quality cells for analysis. Data were normalized and pooled, followed by unsupervised clustering with Seurat V4.0, identifying 30 distinct cell clusters (Fig. 1B and Supplementary Fig. 1A). We successfully identified 11 main cell types: including T cells (C0, C1, C2, C5, C8, C11, C14 and C18), B cells (C9 and C24), plasma cells (C22), epithelial cells (C3, C7, C10, C17, C23 and C28), tumor cells (C13), fibrocytes (C4, C19 and C20), macrophages (C6, C15 and C16), dendritic cells (C21), endothelial cells (C12), mast cells (C26) and a small population of goblet cells (C25) (Fig. 1B and C). Clusters C27 and C29 were excluded due to mixed cell types (Supplementary Fig. 1A). Differentially expressed genes and canonical markers confirmed cell identities (Fig. 1D, E), and CNV analysis distinguished tumor cells (Supplementary Fig. 1B). All cell types were derived from multiple samples, indicating classification by cell type rather than patient specificity (Fig. 1F). SD tumors had more epithelial cells and fewer T and B cells compared to PR and surgical tumors. Tumor cells represented 1% and 7% in PR and SD patients, respectively, with none detected in surgical patients. This was possibly due to the early stage of the tumor and the limited resolution of single-cell sequencing (Fig. 1G).

### Clustering and subtype analyses of T cells and NK cells

**Fig. 2:**
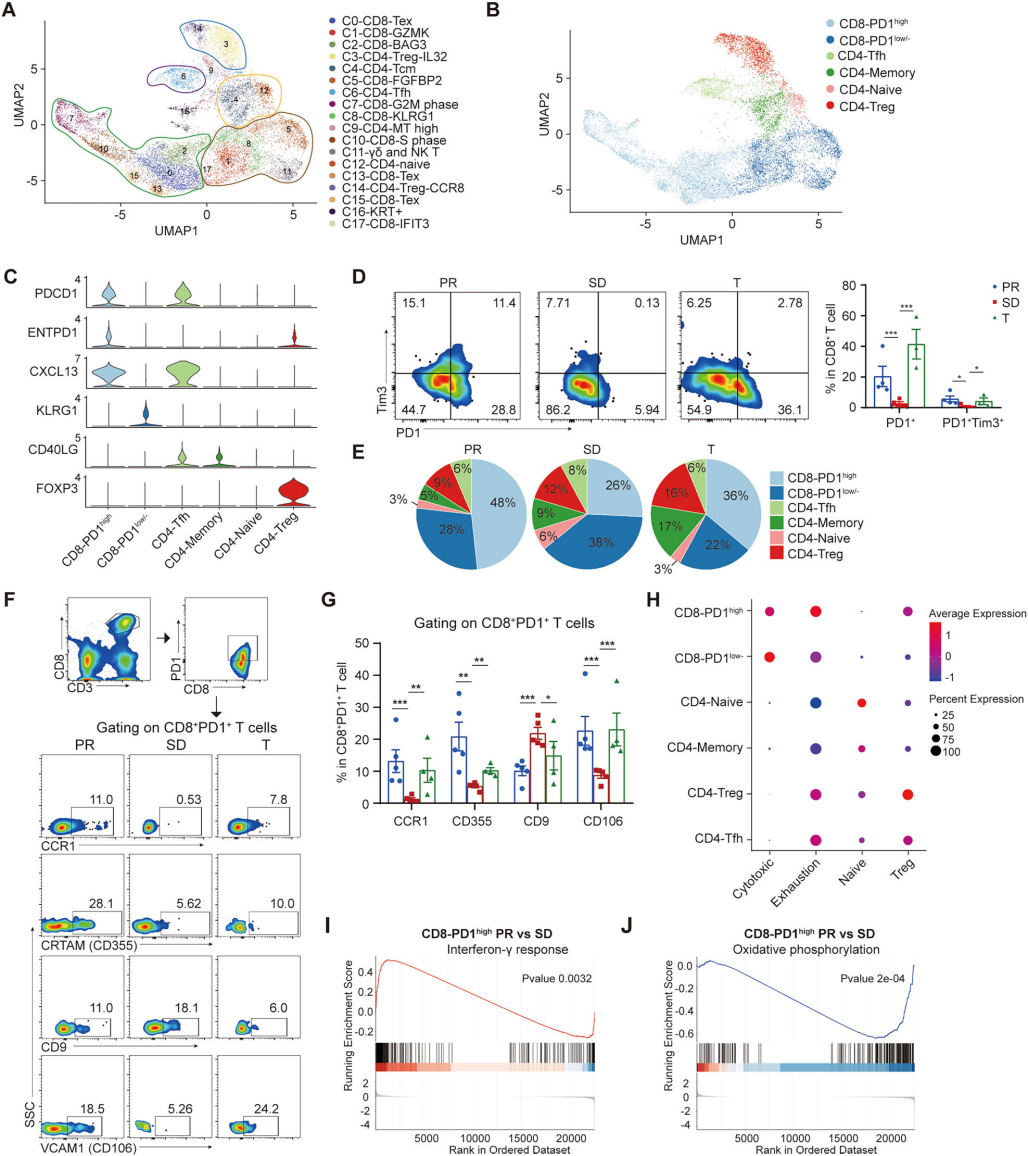
Overview of ESCC-infiltrated T cells. **A** UMAP plot showing ESCC-infiltrated T cells, colored by sub-clusters. **B** UMAP plot showing ESCC-infiltrated T cells colored by main cell types. **C** Violin plots presenting the expression of marker genes in the identified main cell types. **D** Flow cytometry plots depicting PD1 and Tim3 expressions in CD8^+^ T cells sourced from different samples. **E** Pie chart showing the proportion of each main T cell type within the sample source. **F**, **G** Representative flow cytometry plots illustrating the gating strategy for CD8^+^PD1^+^ T cells and the expressions of CCR1, CD355, CD9, and CD106 across PR, SD and surgical tumors. **H** Dot plots showing the enrichment scores of each gene set across various T cell types. **I** GSEA comparing CD8⁺PD1^high^ T cells between PR and SD groups, showing enrichment of the interferon-γ response pathway (*P* = 0.0032). **J** GSEA comparing CD8⁺PD1^high^ T cells between PR and SD groups, showing enrichment of the oxidative phosphorylation pathway (*P* = 2e-04). All experiments were performed at least three times independently with similar results. Statistical significance between two groups was evaluated using Student’s t-tests, while one-way ANOVA followed by Tukey’s post-hoc tests was used for multiple group comparisons (*P < 0.05, **P < 0.01, ***P < 0.001). Values are expressed as mean ± standard error.

T lineage cells emerged as the most prevalent immune cell type. We extracted T and NK cells for unsupervised re-clustering, identifying 18 distinct subclusters (Fig. 2A). A UMAP plot demonstrated their distribution across sample origins (Supplementary Fig. 2A). Based on spatial stratification patterns and differentially expressed genes (DEGs), we categorized CD4 T cell subclusters into central memory (C4), naïve (C12), follicular helper T (Tfh) cells (C6), and two Treg types (C3-CCR8 and C14-IL32) (Supplementary Fig. 2B, C). Three subpopulations (C0, C13, C15) expressed high levels of checkpoint molecules, like PDCD1, HAVCR2, and LAG3, indicating an exhausted phenotype (Supplementary Fig. 2B, D). Additional subclusters, such as C1, C5, C8, and C17, were marked by cytotoxic gene expression (e.g., PRF1, GNLY, NKG7 and KLRG1) (Supplementary Fig. 2B, D). C7 cells showed markers of mitotic division (G2M phase), while C10 cells, enriched in exhaustion genes, exhibited an S phase signature, suggesting exhausted T cells as major intra-tumoral proliferative immune cells [19] (Supplementary Fig. 2B, E). C2 was characterized by stress-related heat shock proteins (e.g., BAG3, HSPA1B and HSPA1A) [20], and C11 featured high expression of markers (e.g., FCER1G, TRDC, KLRC1 and NCAM1) indicative of γδT and NK cells (Supplementary Fig. 2B). Clusters 9 and 16 were deemed low-quality due to high mitochondrial content (Supplementary Fig. 2F) and were excluded from further analysis.

### Altered status of PD1^high^ and PD1^low/−^ T cells in tumors

PD1 expression is crucial for targeting the PD1-PDL1 signaling axis therapeutically. We investigated the composition of PD1^+^ CD8^+^ T cells among PR, SD, and surgical patients by categorizing CD8^+^ T cells into PD1^high^ and PD1^low/−^ groups (Fig. 2B). PD1^high^ CD8^+^ T cells exhibited high expressions of PDCD1, ENTPD1, and CXCL13, whereas PD1^low/−^ cells showed elevated KLRG1 expression (Fig. 2C). Flow cytometry confirmed lower percentages of PD1^+^ and PD1^+^TIM3^+^ CD8^+^ T cells in SD patients relative to PR and surgical patients (Fig. 2D). PD1^high^ CD8^+^ T cells comprised 48%, 26%, and 36% of total T cells in PR, SD, and surgical patients, respectively. PD1^low/−^ CD8^+^ T cells accounted for 28%, 38%, and 22% in the same groups, indicating fewer PD1^high^ cells in SD patients and suggesting an immunosuppressive environment (Fig. 2E). Treg cells were relatively lower in PR (~9%) compared to SD (~12%) and surgical (~16%) patients. Tfh cell proportions remained consistent (~6–8%) across groups. CD4 memory T cells constituted 5%, 9%, and 17% of tumor-infiltrating T cells in PR, SD, and surgical patients, respectively, suggesting that neoadjuvant therapy might encourage naïve CD4 T cell differentiation (Fig. 2E). By focusing on the gating of CD8^+^PD1^+^ T cells, we observed that these cells within SD tumors exhibit low expression levels of CCR1, CD355, and CD106, yet display high expression of CD9. Typically, these molecules are implicated in processes such as immune cell localization, activation, and effector function. Our findings suggest that the migratory and inflammatory responses of CD8^+^PD1^+^ T cells infiltrating SD tumors differ significantly from those observed in PR tumors. (Fig. 2F, G).

Visualization of the exhaustion and cytotoxic scores showed that PD1^high^ CD8^+^ T cells had the highest exhaustion scores, while PD1^low/−^ CD8^+^ T cells exhibited the highest cytotoxic scores (Fig. 2H). Despite their exhaustion profile, PD1^high^ CD8^+^ T cells demonstrated significant cytotoxic potential (Fig. 2H). Notably, PD1^high^ CD8^+^ T cells in PR and surgical patients displayed greater exhaustion characteristics compared to SD patients, although their cytotoxic signatures were similar across PR and SD groups (Supplementary Fig. 3A, B). This suggests that the tumor microenvironment TMEs in SD patients lacks effective antigen presentation and stimulation, resulting in insufficient CD8^+^ T cell priming and less exhaustion.

Further analysis using gene set enrichment analysis (GSEA) on differentially expressed genes in PD1^high^ CD8^+^ T cells between PR and SD patients indicated a heightened Interferon-γ response (Fig. 2I) and a significant downregulation of the Oxidative Phosphorylation pathway (Fig. 2J) in PR patients. These findings reinforce the notion that PD1^high^ CD8^+^ T cells in the SD TMEs are less effectively primed, highlighting the role of the TMEs in modulating immune responses.

### Macrophage in the tumor microenvironment and their compositional differences between sample origins

**Fig. 3:**
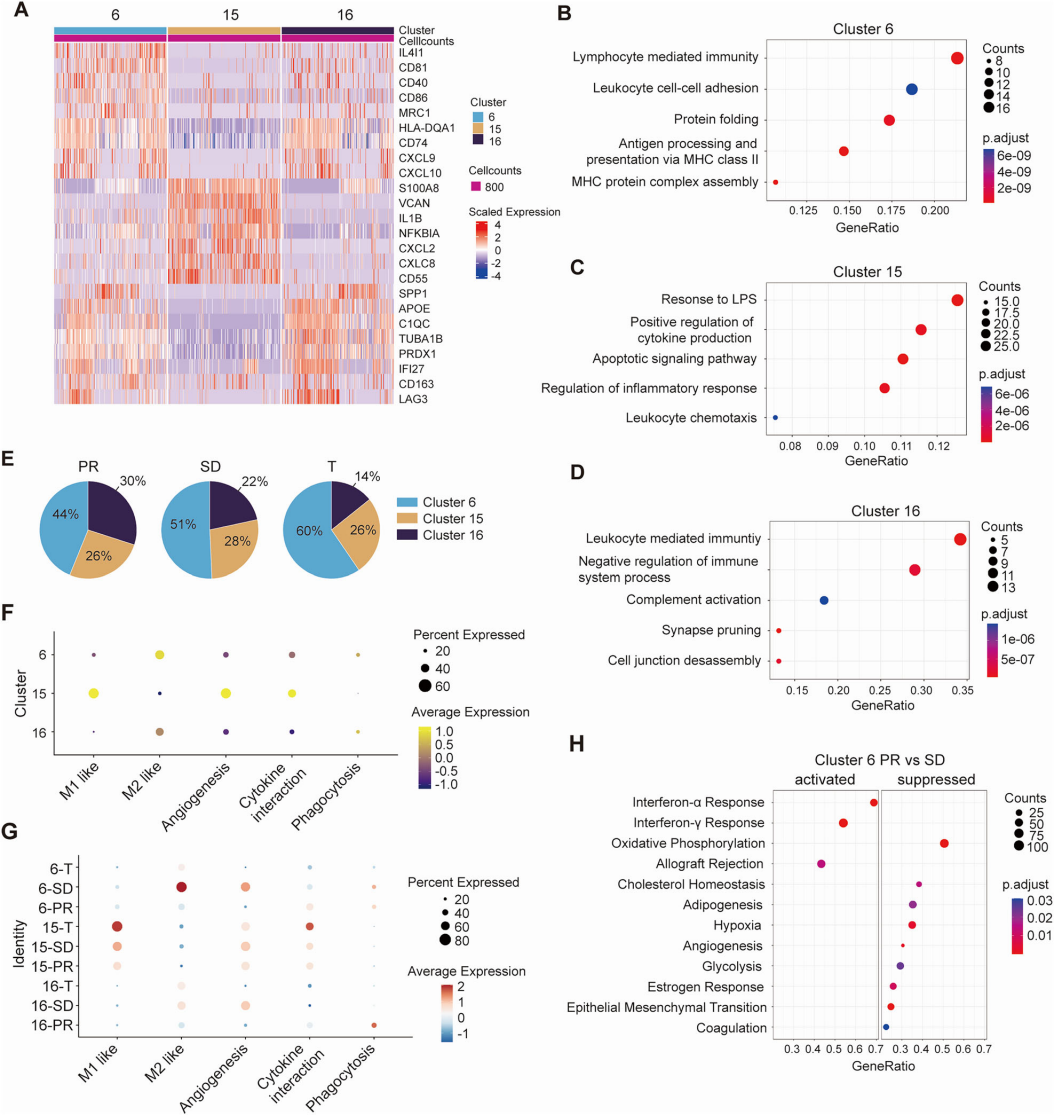
Overview of ESCC-infiltrated macrophages characterized by scRNA-seq. **A** Heatmap displaying the expression of marker genes within each macrophage cluster. **B**–**D** Dot plots showing the enriched pathways across different macrophage clusters. **E** Pie chart showing the proportion of each macrophage cluster derived from the sample source. **F** Dot plots depicting the enrichment scores of each gene set across various macrophage sub-clusters. **G** Dot plots comparing the enrichment scores of each gene set across macrophage sub-clusters originating from PR, SD, and surgical tumors. **H** Dot plots highlighting the differentially enriched pathways in cluster 6 macrophages between PR and SD tumors.

Cell-cell communication via ligand-receptor interactions plays a crucial role in shaping the tumor microenvironment and influencing therapeutic responses. We investigated the interactions between PD1^high^ CD8^+^ T cells and various cell types within the TMEs. Our findings revealed that PD1^high^CD8^+^ T cells most frequently engage with macrophages in the TMEs (Supplementary Fig. 3C). Three subpopulations of macrophages (C6, C15 and C16) were identified (Fig. 1B). C6 macrophages expressed CD81, CD40, CD86 and major histocompatibility complex II (MHC-II) genes such as CD74 and HLA-DQA1 (Fig. 3A). Gene ontology (GO) analysis revealed enrichment of terms related to leukocyte-mediated immunity and antigen presentation via MHC class II, indicating C6 macrophages had antigen-presenting properties (Fig. 3B). C15 macrophages demonstrated high expression levels of S100A8, IL1B, CXCL8, and other inflammation-associated genes (Fig. 3A), with GO analysis highlighting significant enrichment in response to lipopolysaccharide, cytokine production, and leukocyte chemotaxis, suggesting their involvement in inflammatory processes (Fig. 3C). The C16 macrophage highly expressed APOE, C1QC, PRDX1, and IFI27 (Fig. 3A), with enriched GO terms pertaining to leukocyte-mediated immunity and negative regulation of immune system processes (Fig. 3D). C6 macrophages constituted 44%, 51%, and 60% of total macrophages in PR, SD, and surgical patients, respectively. C15 macrophages maintained similar proportions (~26–28%) across samples, whereas C16 macrophages were most prevalent in PR patients (Fig. 3E).

C6 and C16 macrophages were identified as M2 macrophages with phagocytic properties, whereas C15 macrophages exhibited high expression of M1-like, angiogenesis, and cytokine interaction gene sets (Fig. 3F). Notably, C6 macrophages in SD tumors showed the greatest immunosuppressive M2-like gene set expression, alongside relatively higher angiogenesis signatures (Fig. 3G). Chemokines CXCL9 and CXCL10, which recruit Th1 cells, were particularly expressed in cluster-6 and -16 macrophages and were more enriched in PR and surgical tumors compared to SD tumors (Supplementary Fig. 3D). In C6 and C16 macrophages, PR tumors enriched Interferon-α and Interferon-γ responses, while SD tumors had higher oxidative phosphorylation and hypoxia gene expression (Fig. 3H and Supplementary Fig. 3E). Overall, our findings suggested that macrophages infiltrating SD tumors might negatively impact the outcomes of neoadjuvant therapy.

### ISCU regulates macrophage polarization and inflammatory response

**Fig. 4:**
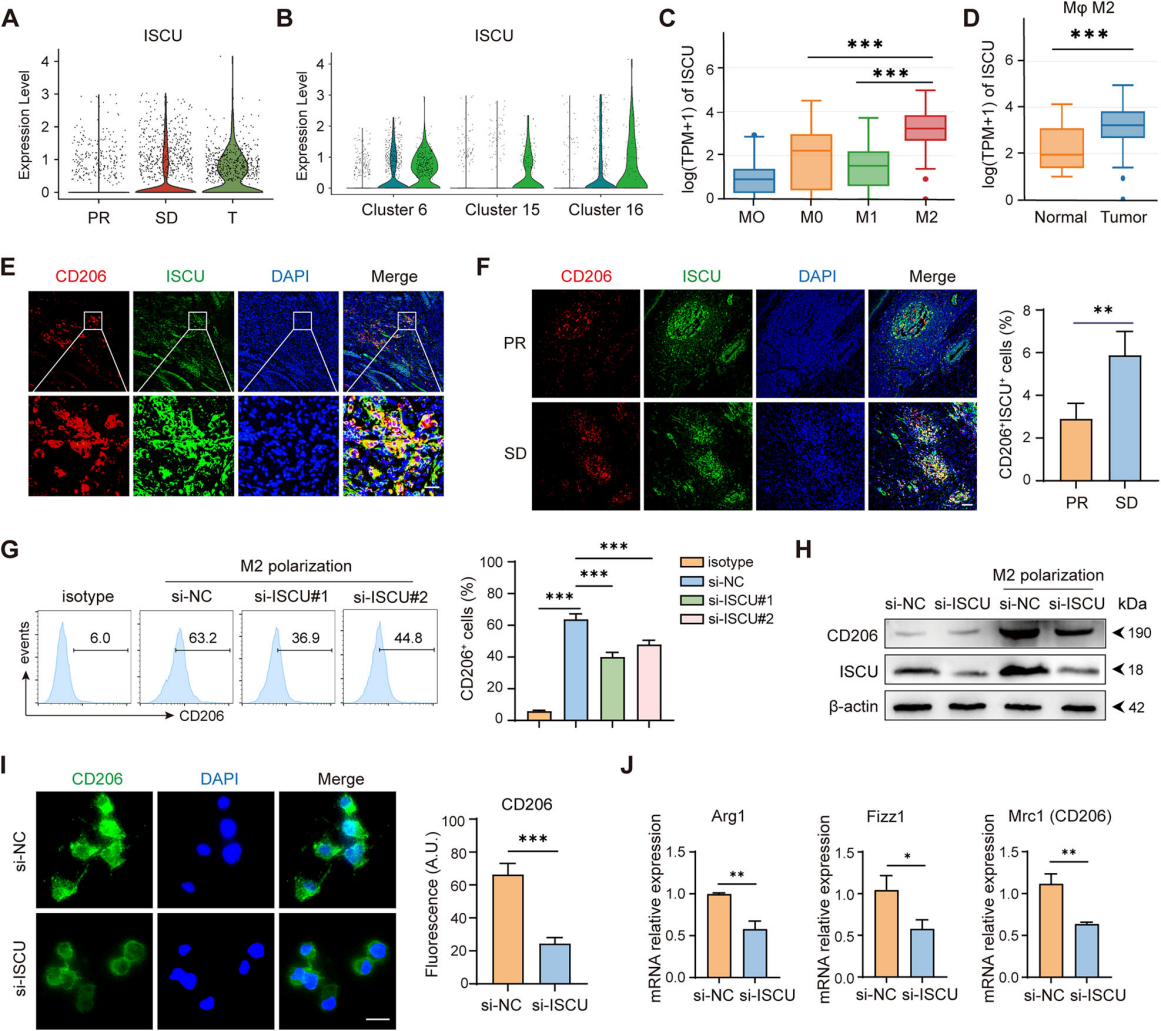
ISCU Regulates macrophage polarization and inflammatory response. **A** Violin plot depicting the expression levels of ISCU across three groups: PR, SD, and T. **B** Violin plot illustrating the distribution of ISCU expression levels for the PR, SD, and T groups within different clusters (Cluster 6, Cluster 15, and Cluster 16). **C** Analysis of ISCU expression levels in different macrophage types within ESCC using GEPIA2021. **D** Comparison of ISCU expression in M2 macrophages between ESCC and normal esophageal tissues via GEPIA2021. **E** Representative immunofluorescence image illustrating ESCC tissue (*n* = 5) co-expressing CD206 and ISCU. Scale bars: 10 μm. **F** Immunofluorescence staining of PR (*n* = 5) and SD (*n* = 5) ESCC tumor samples, highlighting the percentage of CD206^+^ISCU^+^ macrophages. Scale bar: 100 μm. **G** Flow cytometry analysis of CD206 expression in macrophages transfected with si-NC (negative control), si-ISCU#1, or si-ISCU#2 under M2 polarization conditions. The percentage of CD206⁺ cells is shown in the histograms (left), and the quantification is presented as a bar graph (right). **H** Western blot analysis of CD206 and ISCU protein levels in macrophages transfected with si-NC or si-ISCU. **I** Immunofluorescence images indicating CD206 expression in RAW264.7 macrophages transfected with si-NC and si-ISCU. Scale bars: 10 μm. **J** Quantitative RT-PCR analysis of M2 polarization marker gene expressions, including Arg1, Fizz1, and Mrc1, in macrophages following si-NC and si-ISCU transfection. All experiments were performed at least three times independently with similar results. Statistical significance between two groups was evaluated using Student’s t-tests, while one-way ANOVA followed by Tukey’s post-hoc tests was used for multiple group comparisons (*P < 0.05, **P < 0.01, ***P < 0.001). Values are expressed as mean ± standard error.

Comparing DEGs across macrophage subpopulations in PR, SD, and surgical patients, we found that ISCU was highly expressed in SD tumor-infiltrating macrophages but significantly reduced following neoadjuvant therapy (Fig. 4A). Moreover, ISCU was particularly expressed in the C6 and C16-macophages that exhibited the greatest M2-like characteristics (Fig. 4B). Using public databases, we observed that immunotherapy reduces ISCU expression in macrophages (Supplementary Fig. 4A, B) and identified a significant upregulation of ISCU expression in M2-like macrophages (Fig. 4C, Supplementary Fig. 4C, D), especially in tumor infiltrated M2-like macrophages (Fig. 4D). Correspondingly, CD206 and ISCU are co-expressed in esophageal cancer tumor sections (Fig. 4E). Moreover, the proportion of CD206^+^ISCU^+^ M2 macrophages infiltrating SD tumors is significantly greater than that in PR tumors (Fig. 4F). We further investigated whether ISCU influences macrophage polarization. Following M2 polarization stimulation, CD206 expression in Raw264.7 cells significantly increased, whereas ISCU knockdown impaired M2-like polarization (Fig. 4G–I). The knockdown efficiencies of ISCU and p53 were shown in Supplementary Fig. 4E–G. This was corroborated by changes in M2-related biomarkers, including CD206, Arg1 and Fizz1, as analyzed by RT-PCR (Fig. 4J). Taken together, ISCU deficiency impairs M2 macrophage polarization, thereby diminishing the anti-inflammatory and tissue remodeling capabilities of M2 macrophages.

### ISCU regulates ferroptosis to promote M2 polarization

**Fig. 5:**
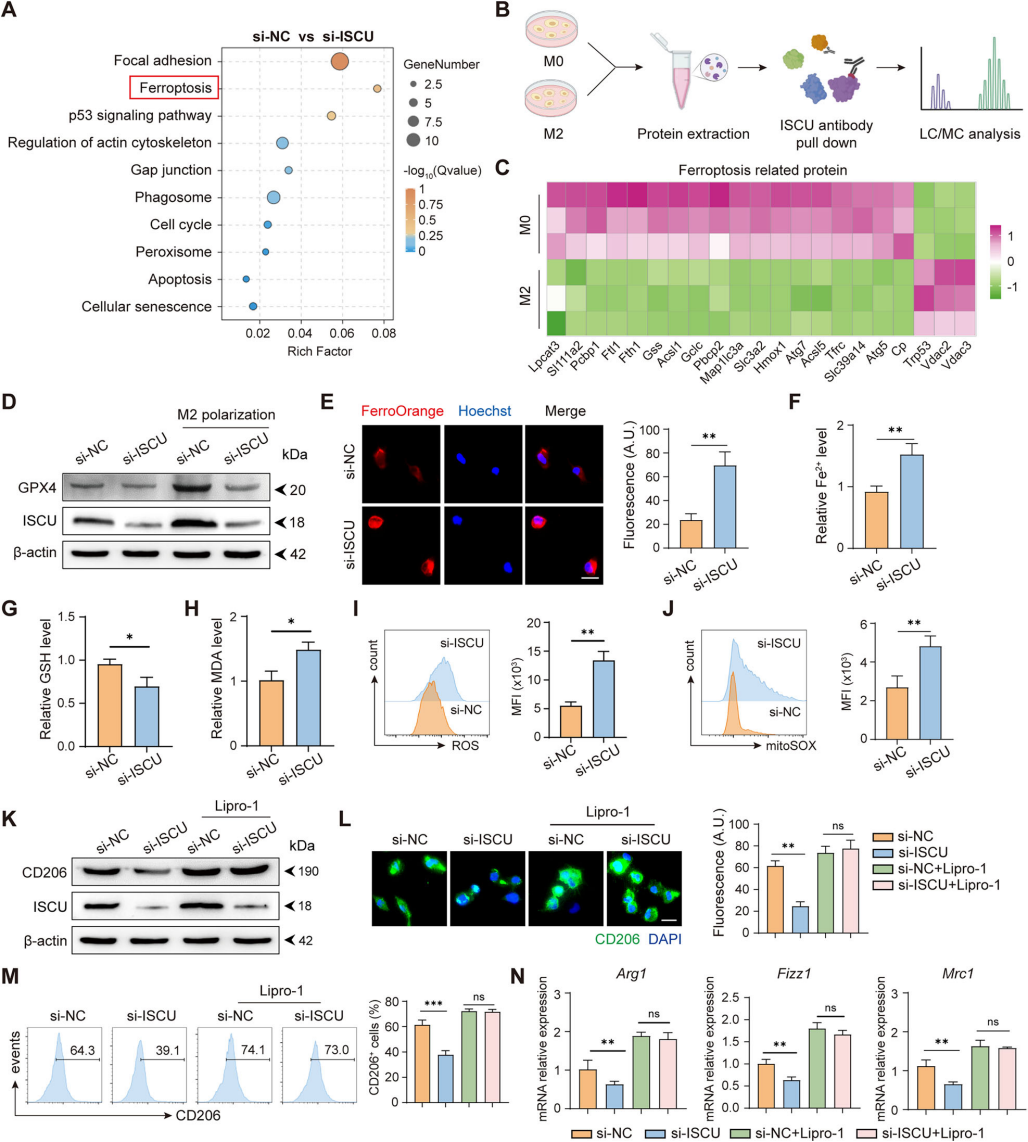
ISCU regulates macrophage polarization through ferroptosis. **A** Pathway enrichment analysis of biological processes in macrophages transfected with si-NC and si-ISCU. **B** Schematic representation of macrophage samples prepared for mass spectrometry analysis. **C** Heatmap showing the interaction of ISCU with ferroptosis-related proteins in M0 and M2 macrophages. **D** Western blot analysis showing GPX4 and ISCU expressions in macrophages transfected with si-NC and si-ISCU. **E** Immunofluorescence imaging indicating Fe²⁺ content in RAW264.7 macrophages transfected with si-NC and si-ISCU. Scale bars: 20 μm. **F** Quantification of intracellular Fe²⁺ levels in si-NC and si-ISCU transfected macrophages. Relative levels of GSH (**G**) and MDA (**H**) in macrophages transfected with si-NC and si-ISCU. Flow cytometry plots showing ROS (**I**) and MitoSOX (**J**) expression in macrophages transfected with si-NC and si-ISCU. **K** Western blot analysis showing CD206 and ISCU expressions in si-NC and si-ISCU transfected macrophages, with and without Lipro-1 treatment. Immunofluorescence images (**L**) and flow cytometry plots (**M**) showing CD206 expression in si-NC and si-ISCU transfected macrophages, with and without Lipro-1 treatment. Scale bars: 10 μm. **N** Quantitative RT-PCR analysis of M2 polarization marker gene expressions, including Arg1, Fizz1, and Mrc1, with and without Lipro-1 treatment. All experiments were performed at least three times independently with similar results. Statistical significance between two groups was evaluated using Student’s t-tests, while one-way ANOVA followed by Tukey’s post-hoc tests was used for multiple group comparisons (ns not significant; *P < 0.05, **P < 0.01). Values are expressed as mean ± standard error.

To investigate the mechanism by which ISCU regulating M2 macrophage polarization and function, we performed RNA bulk sequencing on M2-like Raw264.7 cells transfected with either si-ISCU or si-NC, illustrated by Supplementary Fig. 5A, B. By analyzing altered biological processes, ISCU knockdown cells displayed a significant enrichment in the ferroptosis pathway compared to the si-NC group (Fig. 5A). Subsequent proteomic analysis demonstrated that during macrophage differentiation into the M2 phenotype, ISCU interacts with numerous ferroptosis-related proteins (Fig. 5B, C). Additionally, there is a marked increase in the binding of ISCU with Trp53, Vdac2, and Vdac3 proteins during this differentiation process (Fig. 5C). The RNA-seq and proteomic results collectively suggest that ISCU may regulate macrophage differentiation and function through ferroptosis. GPX4, a vital suppressor of ferroptosis responsible for maintaining lipid redox homeostasis, was found to be downregulated in si-ISCU macrophages during M2 polarization (Fig. 5D). This suggests that ISCU deficiency induces ferroptosis in M2 macrophages. This finding is further corroborated by ferroptosis assays, which show an upregulation of intracellular Fe²⁺ levels following ISCU inhibition (Fig. 5E, F). It is well known that GSH is crucial in preventing ferroptosis by supporting GPX4 in reducing lipid peroxides, while elevated levels of MDA and ROS indicate increased lipid peroxidation and oxidative stress, leading to the onset of ferroptosis. Here, the cellular levels of GSH and MDA, as well as ROS and mitoSOX levels, collectively indicated that ISCU expression maintains a low level of ferroptosis (Fig. 5G–J). To confirm that ISCU regulates M2 polarization through ferroptosis, we applied the ferroptosis-specific inhibitor, Liproxstatin-1. Remarkably, Liproxstatin-1 abolished the decrease in M2 polarization in ISCU-deficient cells, rendering the polarization levels comparable to those in control cells (Fig. 5K–N). In summary, ISCU facilitates the polarization of macrophages towards the M2 phenotype by regulating iron metabolism and actively suppressing ferroptosis.

### ISCU Regulates M2 macrophage polarization in a p53-dependent manner

**Fig. 6:**
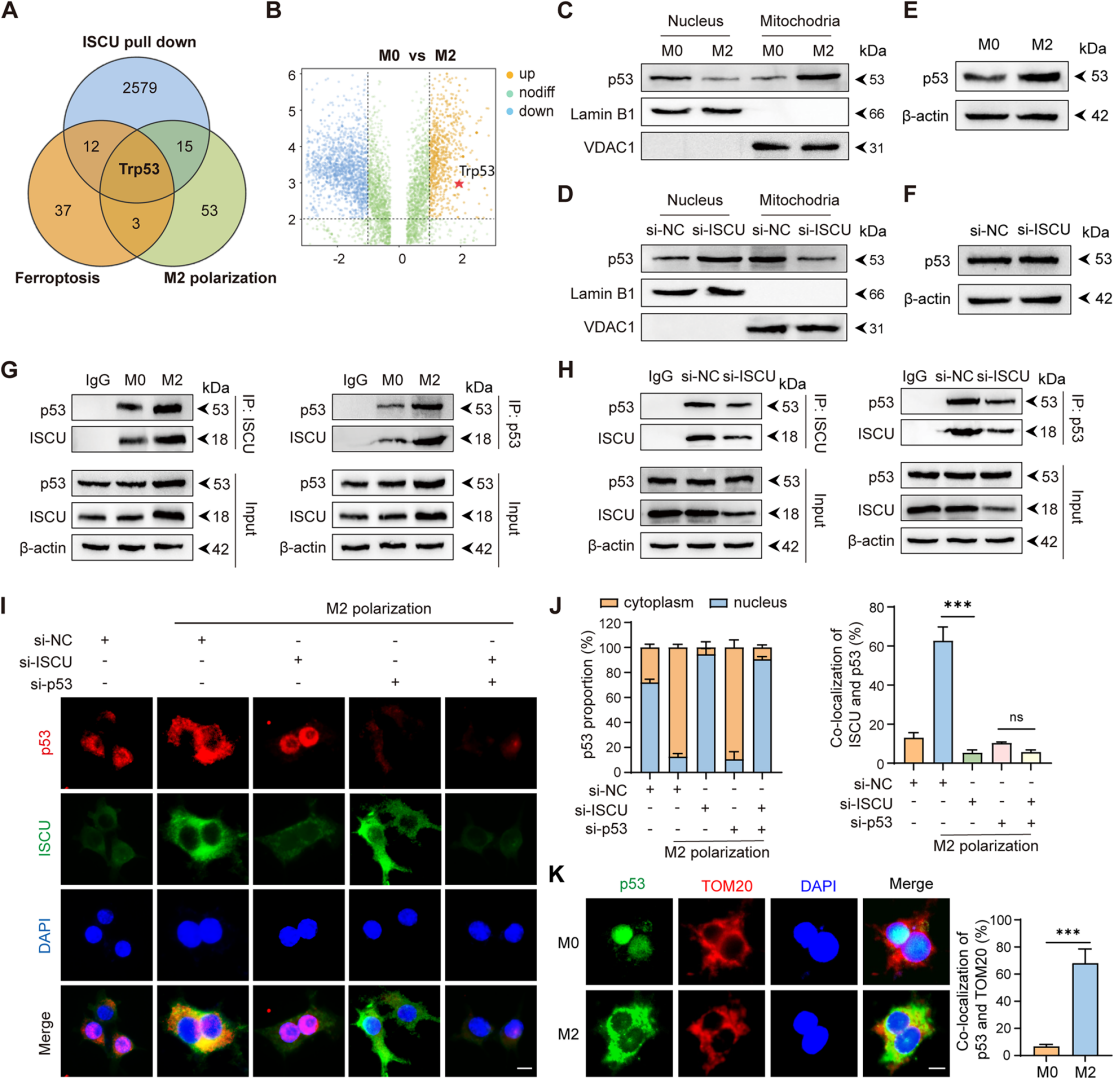
ISCU Binds to the P53 Protein, Preventing Its Translocation from Mitochondria to the Nucleus. **A** Venn diagram depicting overlapping genes among three datasets: ferroptosis-related genes, M2 polarization-related genes, and proteins significantly interacting with ISCU. **B** Volcano plot highlighting p53 as an upregulated interacting protein with ISCU following M2 polarization. **C** Western blot analysis showing p53 levels in mitochondria and nucleus under M0 and M2 culture conditions. **D** Western blot analysis showing p53 levels in mitochondria and nucleus following si-ISCU transfection under M2 macrophage polarization conditions. **E** Western blot analysis showing p53 expression under M0 and M2 culture conditions. **F** Western blot analysis showing p53 levels following si-ISCU transfection under M2 macrophage polarization conditions. **G** Determination of the association between p53 and ISCU via immunoblotting of immunoprecipitates after M2 polarization. **H** Determination of the association between p53 and ISCU via immunoblotting of immunoprecipitates following si-ISCU transfection. **I** Immunofluorescence staining of p53 (red), ISCU (green), and nuclei (DAPI, blue) in macrophages transfected with si-NC, si-ISCU, or si-p53 under M2 polarization conditions. Merged images show colocalization of p53 and ISCU. Scale bar: 10 μm. **J** Quantification of p53 localization in the cytoplasm and nucleus (left) and colocalization of p53 with ISCU (right) under the indicated conditions. **K** Immunofluorescence staining of p53 (green), TOM20 (red), and nuclei (DAPI, blue) under M0 and M2 macrophage polarization conditions. Merged images show colocalization of p53 and TOM20. Quantification of p53 and TOM20 colocalization is shown on the right. Scale bar: 10 μm. All experiments were performed at least three times independently with similar results. Statistical significance between two groups was evaluated using Student’s t-tests, while one-way ANOVA followed by Tukey’s post-hoc tests was used for multiple group comparisons (ns not significant; ***P < 0.001). Values are expressed as mean ± standard error.

Through the analysis of overlapping molecules among three datasets (ISCU-interacting proteins, M2 polarization-associated molecules, and ferroptosis-related molecules), we identified ISCU as a pivotal intersection point among these datasets (Fig. 6A). Furthermore, during macrophage differentiation into the M2 phenotype, the interaction between ISCU and the p53 protein becomes more pronounced (Figs. 5C and 6B). Therefore, ISCU likely regulates M2 polarization in a p53-dependent manner. Given that p53 functions via shuttling between organelles, including the mitochondria and nucleus, we examined its localization. During M2 macrophage polarization, we observed an upregulation of p53 expression in the mitochondria, concomitant with a downregulation in the nucleus (Fig. 6C). Additionally, knockout of ISCU was found to reverse the subcellular distribution of p53 during the differentiation from M0 to M2 phenotype (Fig. 6D). Consistent with previous reports [21], P53 protein levels increase during macrophage polarization toward the M2 phenotype (Fig. 6E), and ISCU knockdown has no significant effect on overall P53 protein expression (Fig. 6F). Co-immunoprecipitation (Co-IP) assays confirmed the physical interaction between ISCU and p53, which was enhanced during M2 polarization but reduced upon ISCU knockdown (Fig. 6G, H). Through immunofluorescence experiments, we further confirmed that during M2 macrophage polarization, p53 translocates from the nucleus to the cytoplasm and interacts with ISCU protein. In contrast, ISCU knockout results in a greater retention of p53 within the nucleus (Fig. 6I, J). Furthermore, in M2 macrophages, p53 translocated from the nucleus colocalizes with TOM20 in the mitochondria (Fig. 6K). Taken together, ISCU plays a critical role in facilitating p53 retention in the mitochondria during M2 macrophage polarization.

**Fig. 7:**
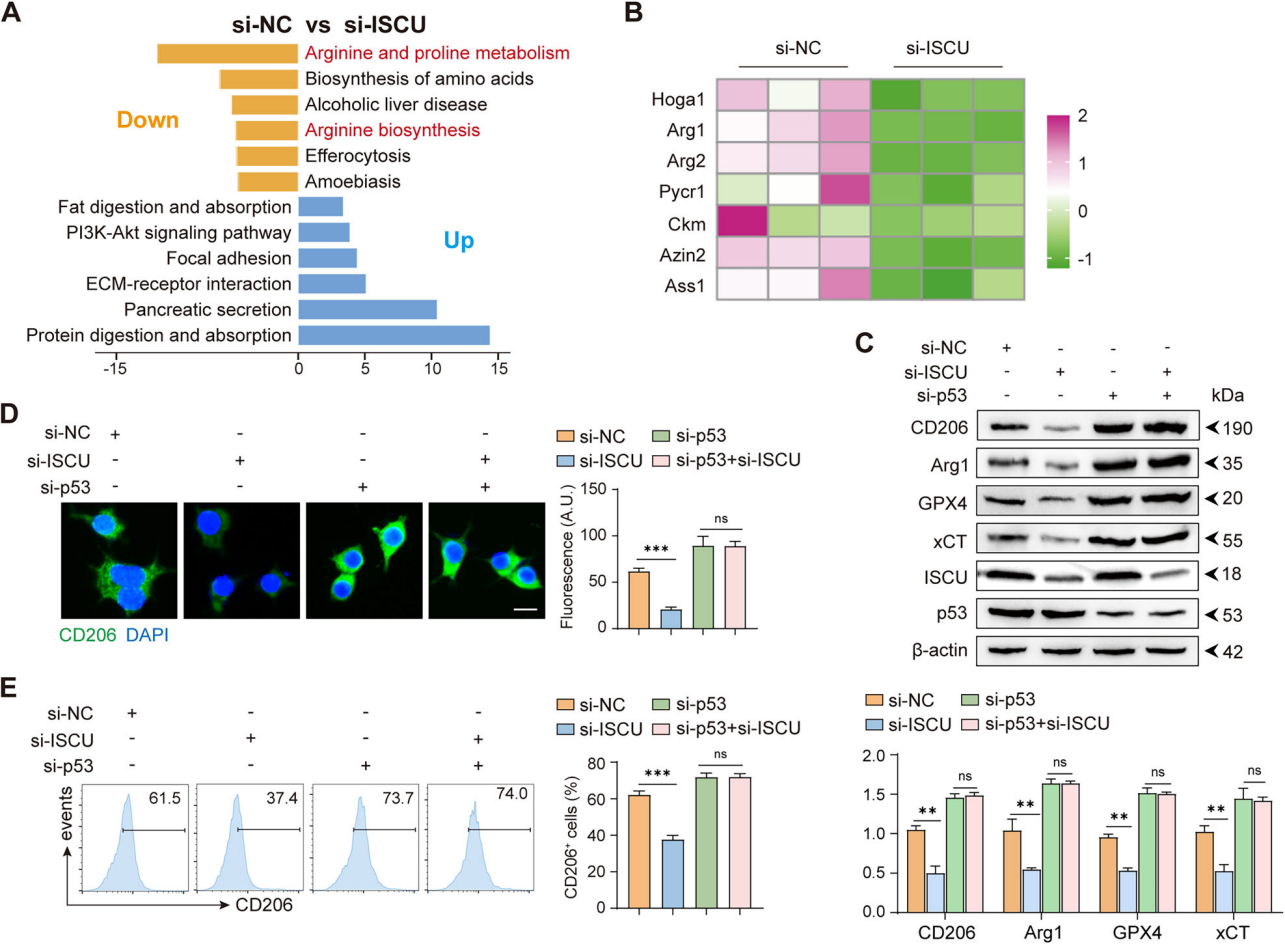
ISCU influenced Arginine metabolism and regulated M2 macrophage polarization in a p53-dependent manner. **A** KEGG pathway enrichment analysis of genes significantly downregulated and upregulated, comparing si-NC with si-ISCU samples using RNA-seq. **B** Heatmap depicting differential gene expression in Arginine and proline metabolism, as well as in Arginine biosynthesis pathways. **C** Western blot analysis showing expressions of CD206, Arg1, GPX4, xCT, ISCU, and p53 following the indicated si-RNAs transfection. Representative blots (top) and quantification of protein levels (bottom) are shown. **D** Immunofluorescence staining of CD206 (green) and nuclei (DAPI, blue) in macrophages transfected with the indicated si-RNA under M2 polarization conditions. Representative images are shown (left), and fluorescence intensity of CD206 is quantified (right). Scale bar: 10 μm. **E** Flow cytometry analysis of CD206 expression in macrophages transfected with the indicated siRNAs under M2 polarization conditions. Quantification of band intensity was performed using ImageJ software. All experiments were performed at least three times independently with similar results. Statistical significance between two groups was evaluated using Student’s t-tests, while one-way ANOVA followed by Tukey’s post-hoc tests was used for multiple group comparisons (ns not significant; **P < 0.01, ***P < 0.001). Values are expressed as mean ± standard error.

P53 is known to transcriptionally downregulate Arg1 expression, thereby suppressing ureagenesis and promoting ammonia elimination in tumor [22]. Our RNA-seq analysis showed that ISCU knockdown significantly suppressed pathways related to arginine and proline metabolism, as well as arginine biosynthesis, prominently affecting the Arg1 gene (Fig. 7A, B). As previously mentioned, p53 negatively regulate the ferroptosis-inhibiting gene xCT [16], thus facilitating the lipid peroxidation that drives ferroptosis. It is hypothesized that ISCU binding to p53 in the mitochondria may inhibit p53’s nuclear translocation and its subsequent transcriptional activity. To test this, we simultaneously knocked down ISCU and p53 in macrophages and observed that the changes in Arg1, GPX4, and xCT expressions induced by ISCU knockdown were abolished (Fig. 7C). Similarly, the simultaneous knockdown of ISCU and p53 also rescued the reduced CD206 expression observed with ISCU knockdown alone (Fig. 7C–E). Consistent with these observations, parallel changes were detected in intracellular Fe²⁺ levels, GSH content, and the lipid peroxidation marker MDA (Supplementary Fig. 5C–F). These results collectively demonstrate that ISCU regulates ferroptosis in macrophages and their M2-polarized phenotype through p53-dependent mechanisms.

### Targeting ISCU in macrophages as a novel anti-tumor therapy

**Fig. 8:**
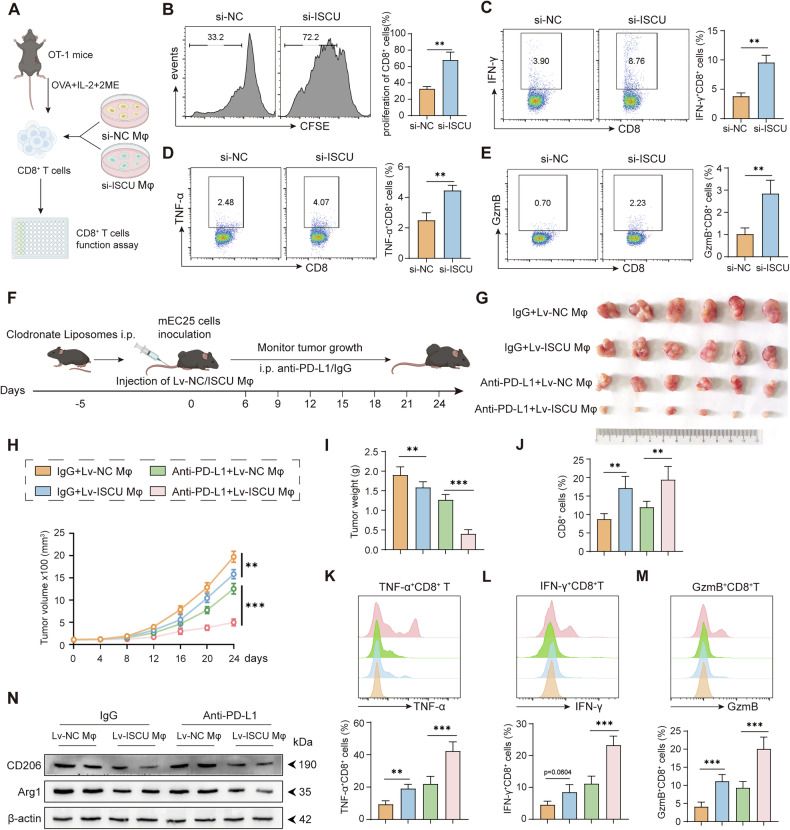
ISCU Inhibition in macrophages enhances anti-tumor immune response. **A** Schematic representation of the antigen-specific CD8^+^ T cell activation assay. Following activation, CD8^+^ T cells were co-cultured with macrophages that had been transfected with or without si-ISCU. The anti-tumor function of the CD8^+^ T cells was then assessed. **B** Flow cytometry plots showing the proliferation ratio of CD8^+^ T cells cultured with si-NC and si-ISCU macrophages. Flow cytometry plots displaying expressions of IFN-γ (**C**), TNF-α (**D**), and GzmB (**E**) in CD8^+^ T cells cultured with control and si-ISCU macrophages. **F** Schematic diagram outlining the construction of the mouse tumor model. Comparison of tumor size (**G**), including tumor volume (**H**) and tumor weight (**I**) in differently treated mice (*n* = 6). **J** CD8^+^ T cell infiltration ratio in variously treated mouse groups (*n* = 6). Flow cytometry plots illustrating expressions of TNF-α (**K**), IFN-γ (**L**), and GzmB (**M**) in CD8^+^ T cells isolated from differently treated mice (*n* = 6). **N** Western blot analysis showing CD206 and Arg1 expressions in mouse tumors. All experiments were performed at least three times independently with similar results. Statistical significance between two groups was evaluated using Student’s t-tests, while one-way ANOVA followed by Tukey’s post-hoc tests was used for multiple group comparisons (**P < 0.01, ***P < 0.001). Values are expressed as mean ± standard error.

Given that ISCU supports and promotes tumor-associated macrophages, we investigated whether ISCU-deficient macrophages could activate CD8^+^ T cells and, subsequently, inhibit tumor growth. According to the experimental scheme detailed in Fig. 8A, ISCU-deficient macrophages significantly enhanced OVA-specific CD8^+^ T cell proliferation (Fig. 8B) and stimulated these T cells to secrete anti-tumor cytokines, including TNF-α, IFN-γ, and Granzyme B (Fig. 8C–E). As outlined in the experimental plan shown in Fig. 8F, these in vivo experiments further validated that ISCU knockout in macrophages led to substantial tumor growth inhibition (Fig. 8G–I) and a reduction in Ki67^+^ tumor cells in mice (Supplementary Fig. 5G), particularly when combined with anti-PD-L1 therapy. The enhanced anti-tumor effect of ISCU-deficient macrophages appeared to be dependent on increased CD8^+^ T cell infiltration (Fig. 8J) and their augmented anti-tumor activity (e.g. TNF-α, IFN-γ and Granzyme B secretions) within the tumor microenvironment (Fig. 8K–M). Consistent with in vivo data, targeting ISCU also reduced CD206 and Arg1 protein expressions in tumor (Fig. 8N), as well as the presence of CD206^+^ macrophage infiltrations in TMEs (Supplementary Fig. 5H). In conclusion, targeting ISCU in macrophages can activate T cell-mediated anti-tumor responses, offering a novel therapeutic strategy against ESCC and potentially other types of cancer.

## Discussion

By sequencing at a single-cell level, we revealed the prominent heterogeneity in distinct cell types in ESCC patients who exhibited different outcomes to combination immunotherapy and chemotherapy. Our findings underscore the immunosuppressive landscape of the SD tumor microenvironment. These immunosuppressive factors within the SD TMEs appear to be intricately linked, promoting tumor survival and progression. Using CellPhoneDB, we identified distinct cellular interactions within the tumor microenvironment, highlighting significant engagement between PD1^high^ CD8^+^ T cells and macrophages. Notably, C6 macrophages derived from SD tumors exhibited the highest M2-like score. GSEA revealed that macrophages infiltrating SD tumors significantly expressed genes associated with oxidative phosphorylation (OXPHOS), adipogenesis, and hypoxia pathways, corroborating the high prevalence of M2-like macrophages in these tumors [23–25]. Differential expression analysis showed that ISCU was significantly upregulated in macrophages derived from SD patients compared to those from PR patients, particularly in the M2-like C6 and C16 macrophage subpopulations. The upregulation of ISCU in the SD tumor microenvironment may be regulated by epigenetic mechanisms, such as miRNA-210 [13], or influenced by iron overload within the TME. Conversely, iron depletion leads to decreased ISCU mRNA levels and reduced protein biosynthesis [26]. We further demonstrated that the inhibition of ISCU significantly disrupted M2 macrophage polarization. Notably, this effect was dependent on ferroptosis, as the application of ferroptosis inhibitors alleviated the observed changes following ISCU inhibition.

Unlike apoptosis, ferroptosis is distinguished by iron-dependent lipid peroxidation and the significant release of inflammatory cytokines, making it inherently more immunogenic [15]. Iron accumulation in macrophages leads to the release of proinflammatory cytokines, such as TNF, and promotes a rapid shift from the M2 to the M1 phenotype [27, 28]. Additionally, ferroptosis contributes to inflammation through the release of damage-associated molecular patterns (DAMPs), which activate immune responses [29]. Conversely, certain inflammatory cytokines, including TNF, PGE2, IL-6, and IL-1, can directly affect GPX4 levels and trigger ferroptosis [30]. Thus, ferroptosis and macrophage inflammatory responses are intricately linked through complex regulatory pathways involving iron metabolism, reactive oxygen species (ROS) production, and cytokine expression. In our study, we demonstrated that ferroptosis in macrophages is significantly associated with their phenotypic transformation and inflammatory response, and that ISCU plays a crucial role in regulating macrophage ferroptosis.

Through the analysis of overlapping molecules among three datasets—ISCU-interacting proteins, M2 polarization-associated molecules, and ferroptosis-related molecules—we identified that p53 at the intersection of these sets. Our data confirm that ISCU interacts with the p53 protein and regulates its translocation between the mitochondria and nucleus during M2 macrophage polarization. The tumor suppressor protein p53 is widely recognized for its role in regulating cell cycle arrest, apoptosis, and genomic stability [31]. Recent studies have expanded its role to include regulation of macrophage inflammatory response and differentiation. Specifically, p53 activation down-regulates M2 gene expression by repressing the transcription of the c-Myc gene [21]. Furthermore, loss of p53 in the myeloid lineage shifts cytokine polarization towards an M1-like phenotype [32]. p53 activation has also been shown to suppress Arg1 expression, an enzyme critical for M2 macrophages [22].

Additionally, p53 also influences iron metabolism and modulates cell sensitively to ferroptosis. For example, p53 down-regulates transferrin receptor 1 levels, and increases ferritin, thereby mimicking the effects of iron chelation and inhibiting cell cycle progression [33]. Conversely, ferroptosis inhibitors can regulate p53 expression [34]. Consistent with this, Liproxstatin-1 treatment reduces p53 levels (Supplementary Fig. 4H), which may explain the slight increase in CD206 and Arg1 expressions in Fig. 5K–N. Another report demonstrated that ISCU is a p53-inducible gene following DNA damage. ISCU inhibits IRP1 binding to iron-responsive elements (IREs) in FTH1 and TFRC, thereby increasing FTH1 translation, reducing TFRC mRNA, and lowering intracellular iron levels [35]. p53 also inhibits cystine uptake and sensitizes cells to ferroptosis by repressing the expression of SLC7A11, a key component of the cystine/glutamate antiporter [16]. Thus, p53 regulates cellular iron homeostasis in response to various stimuli through multiple mechanisms, including the modulation of ISCU expression. In our data, we determined that knocking down p53 expression attenuates the effects of ISCU on macrophage ferroptosis. Mechanistically, ISCU influences the functional activity of p53 by maintaining its retention in the mitochondria and regulating its nuclear translocation. Thus, ISCU interacts with p53 to regulate their expression and spatial distribution, collectively influencing macrophage iron homeostasis, susceptibility to ferroptosis, and inflammatory response. This study also has unresolved issues. Iron-sulfur cluster proteins play critical roles in electron transport, metabolic regulation, and DNA repair, some of which overlap with the functions of p53 [12, 31]. Research on the connection between p53 and ISCU is continuously advancing. The mechanism by which ISCU interacts with the p53 protein, and whether it functions as a co-factor for p53, remains to be studied.

Extensive research efforts have focused on enhancing T cell-mediated antitumor immunity. Importantly, macrophage polarization occurs independently of T cells: M1 macrophages drive Th1 immune responses through IFN-γ secretion, while M2 macrophages promote Th2 responses and Treg cell generation via TGF-β and IL-10 production. This functional dichotomy of macrophages from different sources presents a promising therapeutic window for targeted cancer immunotherapy. In our study, we demonstrated that ISCU deficiency in macrophages markedly enhanced both antigen-specific CD8^+^ T cells proliferation and their cytotoxic cytokine production. The development of ISCU antagonists suitable for in vivo administration could potentially expand the therapeutic applications of this approach.

Taken together, we elucidated the complex composition of immune and non-immune cells within the TMEs and highlighted an immunosuppressive status in SD tumors. We demonstrated that inhibiting ISCU expression in macrophages suppresses M2 macrophage differentiation and the expression of M2-associated genes. This mechanism involves ISCU blocking the translocation of p53 from the mitochondria to the nucleus, thereby inhibiting the expression of downstream p53 target genes, including those related to ferroptosis and Arg1-regulated metabolism pathways. Thus, ISCU could serve as a potential target for enhancing immune checkpoint inhibition therapy in ESCC patients.

## Materials and methods

### Human specimens

Human tissue specimens for sc-RNA sequencing were obtained from five ESCC patients who underwent surgery at Guangdong Provincial People’s Hospital (Guangzhou, China), with informed written consent. Detailed clinical information of the patients is provided in Supplementary Table 1. All samples in this study were approved by the Committees for the Ethical Review of Research Involving Human Subjects at the Guangdong Provincial People’s Hospital.

### Single-cell collection and sorting

Tumor samples were minced into small pieces and digested with collagenase type IV (0.5 mg/ml) and DNase I (50 μg/ml) in RPMI-1640 medium (Invitrogen) for 30 min at 37 °C. The digested tumor pieces were gently triturated using a 10-ml syringe plunger through a 70-μm cell strainer (BD Biosciences) in RPMI-1640 medium supplemented with 10% FBS until uniform cell suspensions were obtained. Subsequently, the cell suspensions were incubated in 1× red blood cell lysis buffer (BD Biosciences) on ice for 5 min. Dead cells were removed from the tissue preparations using the Dead Cell Removal Kit (Miltenyi Biotec). Viable cells were examined and counted before being subjected to sequencing using the 10x Genomics platform. scRNA-seq libraries were constructed as previously described [36].

### Single-cell RNA-seq data processing

The single-cell RNA-seq data were processed using Cell Ranger software (v.3.0.2) provided by 10x Genomics, with default parameters. The reads were aligned to the GRCh38 reference genome, and raw gene expression metrics were generated for each sample. For quality filtering, genes detected in less than 0.1% of all cells were removed, and cells with fewer than 500 gene counts or with mitochondrial RNA content exceeding 20% were filtered out. Highly variable genes were selected based on average expression and dispersion thresholds using the FindVariableGenes function with default settings. Normalized expression levels for each gene were adjusted for the total UMI counts and mitochondrial RNA content per cell using the ScaleData function. Principal component analysis (PCA) was then conducted using the RunPCA function. Graph-based Louvain clustering was performed on the top 20 principal components (PCs) using the FindClusters function, with the resolution parameter set to 0.8. The clusters were visualized using the Uniform Manifold Approximation and Projection (UMAP) method. Cluster-specific marker genes were identified with the Wilcoxon test, implemented in the FindAllMarkers function. Manual annotation of cell clusters was based on these identified marker genes.

### Copy number variation analysis

Copy number variations (CNVs) in tumor cells were estimated from the scRNA-seq results using expression levels, following referenced methodologies. CNV analysis was conducted using the dataset GTEx_8_tissues_snRNAseq_atlas_071421.public_obs.h5ad, available for download from the GTEx Portal at https://www.gtexportal.org/home/datasets. Our analysis specifically focused on the epithelial cells in the esophagus mucosal tissue, with a random selection of 1000 cells for detailed examination. The calculation method was adapted from Patel et al., Science 2014 [37], with modifications in the gene selection approach. Specifically, we calculated the average expression for each gene and retained only those genes with expression levels greater than the mean.

### Analysis of gene set variation

Gene ontology (GO) and Kyoto Encyclopedia of Genes and Genomes (KEGG) analyses were conducted using the “clusterProfiler” package in R [38]. Pathway analysis was performed based on the 50 hallmarks from the MSigDB database [39]. The pathway activity of individual cells was estimated using the Gene Set Variation Analysis (GSVA) package with standard settings with standard settings [40]. To assess differential pathway activities between program cells and non-program cells, or among different cell subtypes, activity scores for each cell group were compared using the Limma package [41].

### Cell communication analysis

Cell communication analysis was performed using CellPhoneDB, which features a comprehensive database of known ligand-receptor pairs to predict cell-cell interactions [36]. Interaction pairs were considered significant if they had a P-value less than 0.05 and an average log (fold change) greater than 0.1.

### Reagents

Antibodies against CD206 (24595), xCT (98051), GPX4 (59735) and p53 (2524) were purchased from Cell Signaling Technology. The anti-Arg1 (16001-1-AP), anti-ISCU (14812-1-AP), anti-β-actin (66009-1-Ig), anti-Lamin B1 (12987-1-AP), TOM20 (11802-1-AP) and anti-VDAC1 (66345-1-Ig) antibodies were bought from Proteintech. IL-4 (214-14) was obtained from Peprotech. Cytometry antibodies against CD8 (344721), CD45 (103109), CD3 (100221), CD8 (140404), TNF-α (506308), IFN-γ (505808) and Granzyme-B (372213) were bought from BioLegend. Cytometry antibodies against CD8 (562428), PD1 (564017), Tim3 (565569), CD9 (745827), CD106 (555647), CD355 (558996), CCR1 (557914) were brought from BD Biosciences. Liproxstatin-1 (S7699) was from Selleck. OVA-SIINFEKL peptides (HY-P1489), 2-ME (HY-12033) were from MCE. FerroOrange (F374) was purchased from Dojindo company. Hoechst (C1011) was from Beyotime Biotechnology.

### Animal

C57BL/6J mice were purchased from Experimental Animal Center of Southern Medical University. The mice were housed under controlled conditions: a constant temperature of 19–23 °C, 55 ± 10% humidity, and a 12-h light/dark cycle, with free access to water and commercial feedstuff. All animal experiments were approved by the Welfare and Ethical Committee for Experimental Animal Care of Southern Medical University.

### Macrophage polarization and transfection

Macrophage polarization was conducted using RAW264.7 cells or bone marrow-derived macrophages (BMDMs), which were harvested and cultured as previously described (referencing PMID: 36342281). For M2 polarization, the cells were stimulated with IL-4 (10 ng/mL) for 48 h, while M1 polarization was induced by stimulating the cells with LPS (100 ng/mL) for 48 h. For transfection experiments, RAW264.7 cells or BMDMs were first stimulated with IL-4 (10 ng/mL). Concurrently, the cells were transfected with either si-ISCU or si-NC for 24, 48, and 72 h, depending on the specific experimental requirements.

### RNA sequencing

RNA sequencing (RNA-seq) was conducted by Hangzhou Jingjie Biotechnology Co., Ltd. In brief, cells were lysed to extract total RNA, followed with mRNA fragmentation and subsequent cDNA synthesis to generate RNA-seq library. The library products were then purified and underwent quality control assessments. Sequencing was performed using the NovaSeq 6000 platform (Illumina). Data analysis followed published protocols.

### Poteomics of ISCU interaction proteins

Proteomics analysis was conducted by Hangzhou Jingjie Biotechnology Co., Ltd. Cell lysates were incubated with anti-ISCU antibody, followed by incubation with protein A/G agarose (Santa Cruz Biotechnology) for 4 h. The bound proteins were eluted and subjected to mass spectrometry for protein identification on an EASY-nLC 1200 UPLC system (ThermoFisher Scientific). Tandem mass spectra were searched against Mus_musculus_10090_SP_20231220.fasta (17,191 entries) concatenated with reverse decoy database.

### Immunofluorescence and Immunohistochemistry

Immunofluorescence and immunohistochemistry procedures were carried out as previously described (referencing PMID: 35381393). Briefly, cells or tissue sections were incubated with a blocking solution, followed by overnight incubation with primary antibodies at 4 °C. The samples were then incubated with the corresponding secondary antibodies for 1 h at room temperature.

### Immunoprecipitation and western blot

Whole-cell lysates were prepared using cell lysis buffer for immunoprecipitation (IP) and western blotting (P0013, Beyotime Biotechnology). In some experiments, nuclear (P0027, Beyotime Biotechnology) or mitochondria (C3601, Beyotime Biotechnology) were isolated for further analysis. For the immunoprecipitation assay, total proteins were incubated with 1 μg of the relevant antibodies overnight at 4 °C. Subsequently, protein A/G agarose beads were added, and incubation continued for an additional 4 h at 4 °C. The immunoprecipitants were then eluted and analyzed by SDS-PAGE. For western blot analysis, protein samples were separated by SDS-PAGE and transferred onto PVDF membranes. The membranes were blocked with 5% BSA for 1 h at room temperature, followed by incubation with the indicated primary antibodies overnight at 4 °C. The membranes were then incubated with HRP-conjugated secondary antibodies for 1 h at room temperature.

### Detection of GSH, MDA and Fe^2+^

Following the indicated treatments, cells were collected, lysed, and processed according to the manufacturer’s instructions. Glutathione (GSH, A006-2-1) and malondialdehyde (MDA, A003-4-1) assays were conducted using kits from Nanjing Jiancheng Bioengineering Institute. To detect the intracellular Fe^2+^, cells with indicated treatment were harvested and lysed. Subsequently, cell supernatants were incubated with iron buffer at 37 °C for 30 minutes, followed with measurement by microplate reader (OD = 593 nm). The Fe²⁺ detection kit was obtained from Abcam (ab83366).

### Flow cytometry

Flow cytometry detection method for surface markers of tumor-infiltrating T cells, referring to the original article [42]. For CD206 detection, cells were obtained and stained with anti-CD206 (141708, Biolegend) at 4 °C for 30 min in the dark. 7AAD (A1310, Thermo Fisher Scientific) was used to identify live cells. For the ROS assay, cells were harvested and stained with DCFH-DA (10 μM, S0033S, Beyotime Biotechnology) at 37 °C for 30 min in the dark, followed with wash and flow cytometry analysis. For the MitoSOX assay, cells were harvested and stained with MitoSOX Red (5 μM, S0061S, Beyotime Biotechnology) at 37 °C for 20 min in the dark, followed with wash and flow cytometry analysis.

### RT-qPCR analysis

Total RNA was extracted using 1 mL of TRIzol® reagent (Thermo Fisher Scientific) and subsequently transcribed into cDNA. Quantitative PCR (qPCR) was performed using SYBR Green (A46112, Thermo Fisher Scientific) according to the manufacturer’s instructions. The expression levels of the target genes were normalized to β-actin and calculated using the 2^−∆∆Ct^ method. The primers used for quantitative real-time PCR in this study are listed in Supplementary Table 2.

### Identification of key protein interacting with ISCU

Ferroptosis related genes (score > 3.0) and M2-polarization genes (score > 5.0) were collected from GeneCards. After converting gene abbreviations to account for different species, overlapping molecules among ferroptosis-related genes, M2-polarization genes, and ISCU significant interaction proteins (log2fc > 1 or < −1, FDR < 0.005) were identified using a Venn diagram.

### CD8^+^ T cell proliferation and anti-tumor function assay

For the proliferation assay, splenocytes were harvested from OT-I transgenic mice and labeled with carboxyfluorescein succinimidyl ester (CFSE, Thermo Fisher Scientific). These splenocytes were stimulated with OVA-SIINFEKL peptides (100 nM) in the presence of IL-2 (5 ng/mL) and 2-mercaptoethanol (55 μM), then seeded at a concentration of 5 × 10^4^ cells per well in flat 96-well plates. Bone marrow-derived macrophages (BMDMs) transfected with si-NC or si-ISCU (2 × 10^5^ cells) were added at the start of the activation. Two days after co-culture, OT-I cell proliferation was assessed by CFSE dilution. OT-I cells were gated as CD45⁺CD3⁺CD8⁺ cells.

For the anti-tumor function assay, splenocytes were harvested from OT-I transgenic mice and seeded at a concentration of 5 × 10^4^ cells per well in flat 96-well plates, with stimulation from OVA-SIINFEKL peptides, IL-2, and 2-mercaptoethanol. BMDMs transfected with si-NC or si-ISCU (2 × 10^5^ cells) were added at the beginning of the activation. Two days after co-culture, OT-I cells were treated with a cell stimulation cocktail (plus protein transport inhibitors) (00-4975-93, Thermo Fisher Scientific) 6 h before harvest, and their anti-tumor function was determined by measuring TNF-α, IFN-γ, and Granzyme-B levels. OT-I cells were gated as CD45⁺CD3⁺CD8⁺ cells.

To detect in vivo CD8⁺ T cell function, single-cell suspensions were harvested from mouse tumors according to a previously described protocol (referencing PMID: 37192617). The isolated cells were treated with a cell stimulation cocktail for 6 h, followed by surface staining with CD45, CD3, and CD8 antibodies, and intracellular staining for TNF-α, IFN-γ, and Granzyme-B.

### ESCC tumor mice model

C57BL/6 J male mice (5-6 weeks old) received subcutaneous injections of mEC25 cells (5 × 10^6^ cells per mouse). Macrophages in the mice were eliminated by administering clodronate liposomes (C-002, Liposoma) 5 days prior to the inoculation of mEC25 cells. Bone marrow-derived macrophages (BMDMs) transfected with either Lv-ISCU or Lv-NC (1 × 10^7^ cells per mouse) were injected via the tail vein on day 0. Starting from day 6, mice were intraperitoneally injected every 3 days with either 200 µg control IgG (BE0090, Bio X Cell) or 10 mg/kg anti-PD-L1 (BE0101, Bio X Cell). Tumors were monitored daily and harvested on day 21. Typically, when the tumor volume reaches 2000 mm³ or the maximum diameter reaches 20 mm (in mice), the experiment is terminated, and the animals are euthanized.

### Statistical analysis

All the results were expressed as mean ± standard deviation (SD). The unpaired two-sample Wilcoxon test was applied to compare cell distribution between two groups of cells. Statistical significance between two groups was evaluated using Student’s t-tests, whereas comparisons involving multiple groups were assessed by one-way analysis of variance (ANOVA), followed by the Tukey’s post-hoc tests. *P* value of <0.05 was considered statistically significant.

## Supplementary information

Supplementary figures

Western blots

## Data Availability

All data needed to evaluate the conclusions in the paper are present in the paper and/or the supplementary material. Additional data related to this paper may be requested from the authors.
