# Supplementary material for: ISCU-p53 axis orchestrates macrophage polarization to dictate immunotherapy response in esophageal squamous cell carcinoma
Source: Cell Death Dis. 2025 Jun 20;16(1):462. doi: 10.1038/s41419-025-07787-7 (PMC12181301; doi:10.1038/s41419-025-07787-7)
Supplement: Supplementary file 1 — Supplementary figures [file 41419_2025_7787_MOESM1_ESM.docx]

Supplementary Materials for

**ISCU-p53 Axis Orchestrates Macrophage Polarization to Dictate Immunotherapy Response in Esophageal squamous cell carcinoma**

Jialiang Luo *et al.*

*** Correspondence:** Qingyun Chen Email: [chenqingyun@gdph.org.cn](mailto:chenqingyun@gdph.org.cn)

Guibin Qiao Email: [qiaoguibin@gdph.org.cn](mailto:chenqingyun@gdph.org.cn)

Supplementary Figures


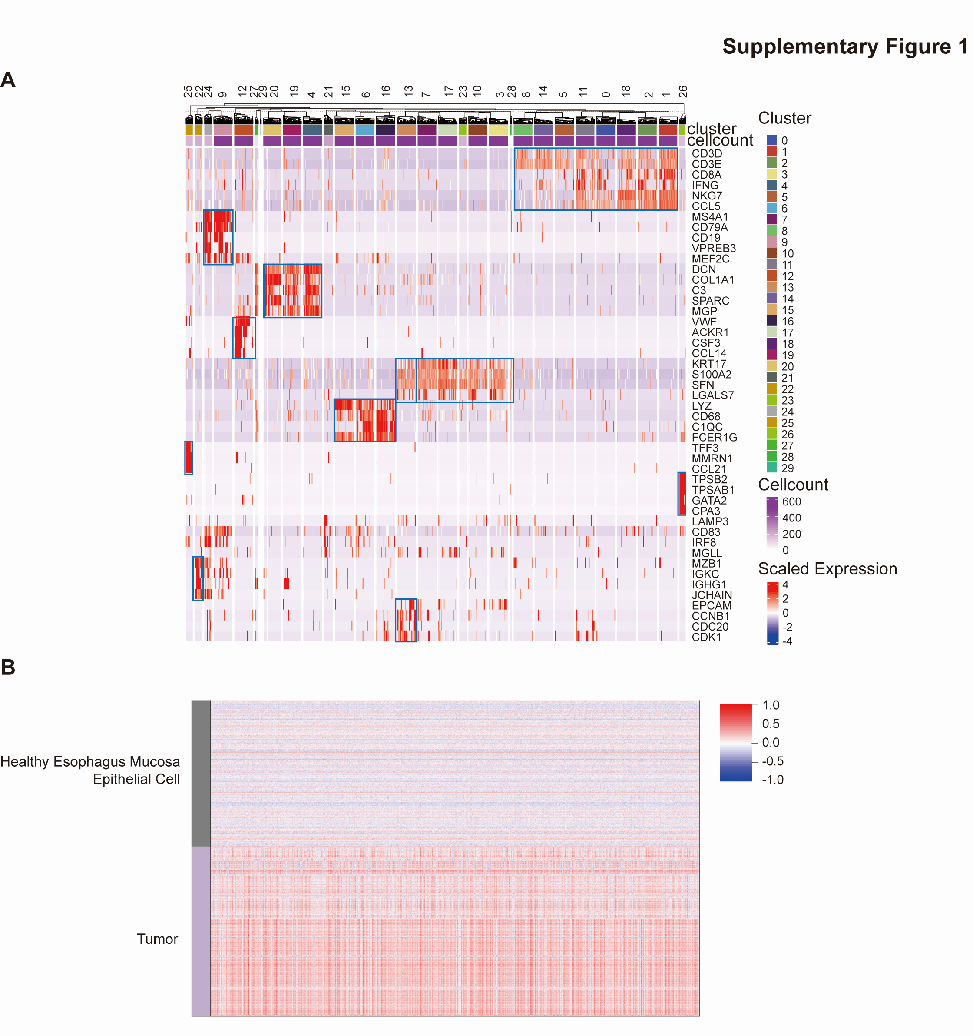


**Fig. S1.**

**A** Heatmap displaying the expression of marker genes in each cell type. **B** Copy number variations (CNVs) specifically focus on the normal epithelial cells within the esophageal mucosal tissue and tumor cells (cluster 13).


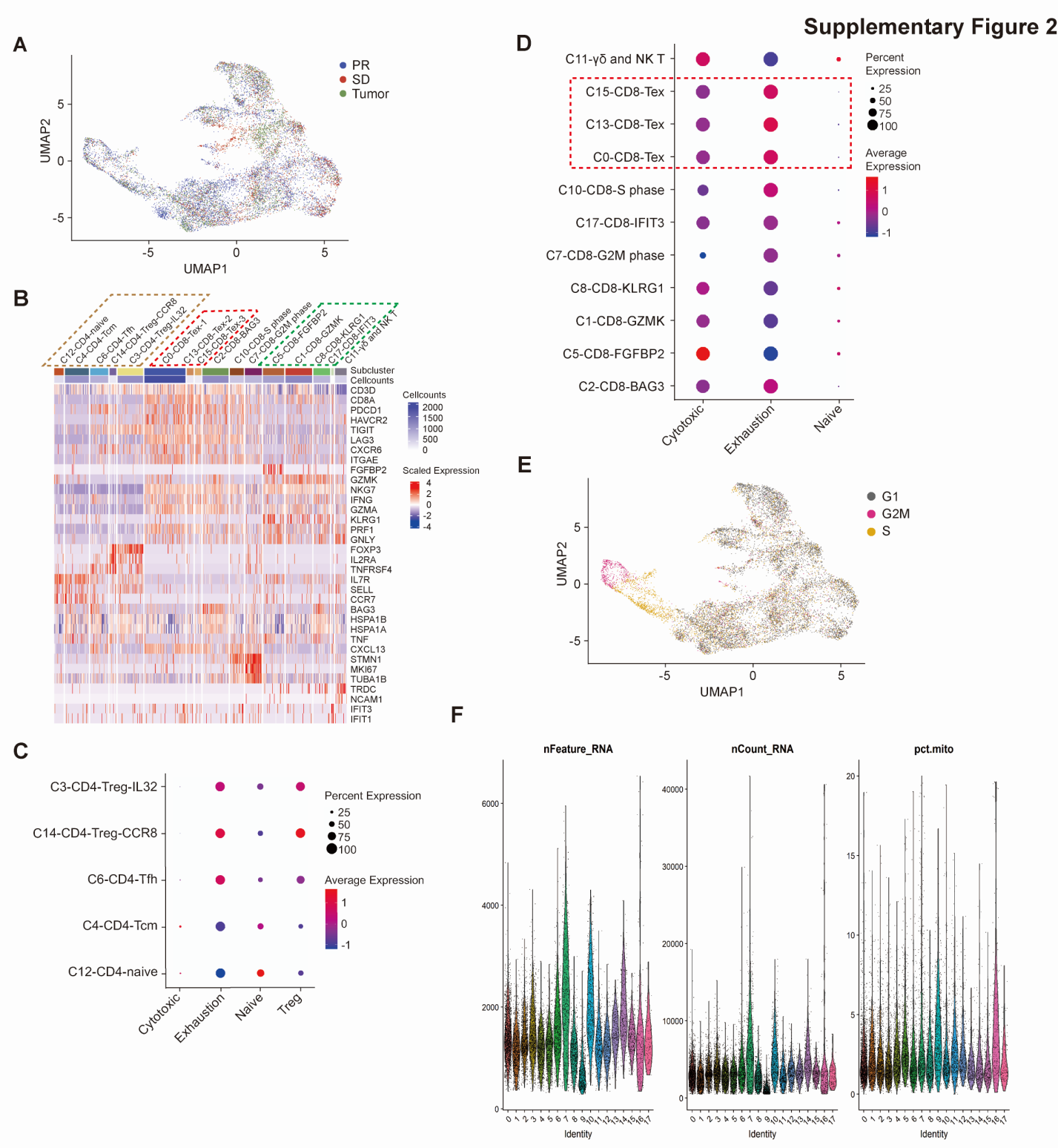
Fig. S2.

**A** UMAP plot showing ESCC-infiltrated T cells, colored by sample source. **B** Heatmap showing the expression of marker genes in each T cell sub-cluster. **C and D** Dot plots displaying the enrichment scores of each gene set across CD4 (C) and CD8 (D) T cell subsets. **E** UMAP plot illustrating the G1/M/G2 phase. **F** Violin plots showing the distribution of nFeature_RNA, nCount_RNA, and percent.mt across different T cell sub-clusters.


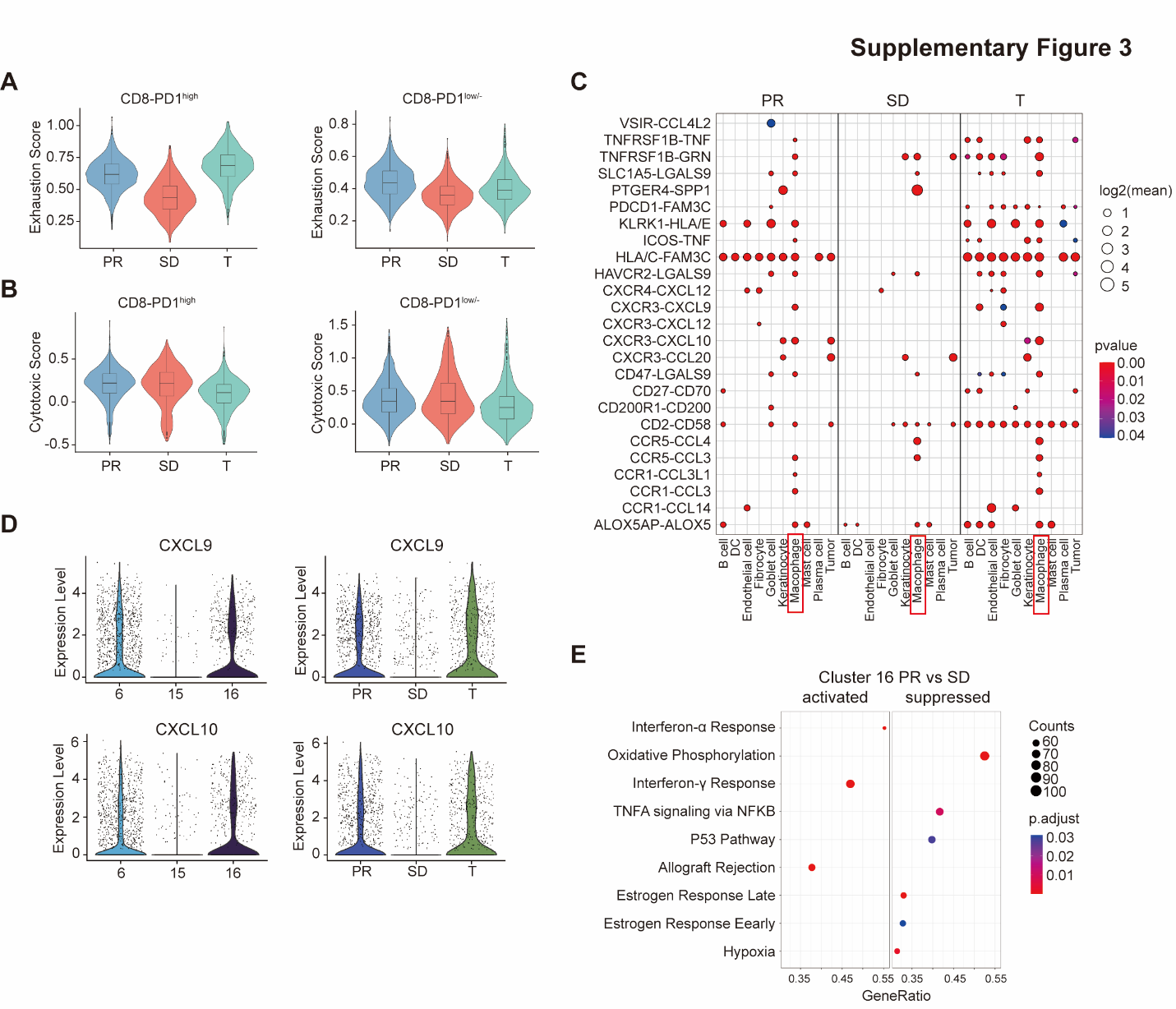


**Fig. S3.**

**A, B** Violin plots illustrating the exhaustion (A) and cytotoxic (B) scores among PD1^high^ and PD1^low/-^ CD8 T cells derived from PR, SD, and surgical tumors. **C** Dot plot generated using CellPhoneDB, highlighting the differentially expressed receptor-ligands interactions among PR, SD, and tumor samples. The size of the dots represents the log_2_(mean) expression, and the color indicates the adjusted p-value. **D** Violin plots displaying the expression levels of CXCL9 and CXCL10 different cell clusters and sample origins. **E** Dot plot showing the enrichment scores of various gene sets in cluster 16, comparing PR and SD samples.


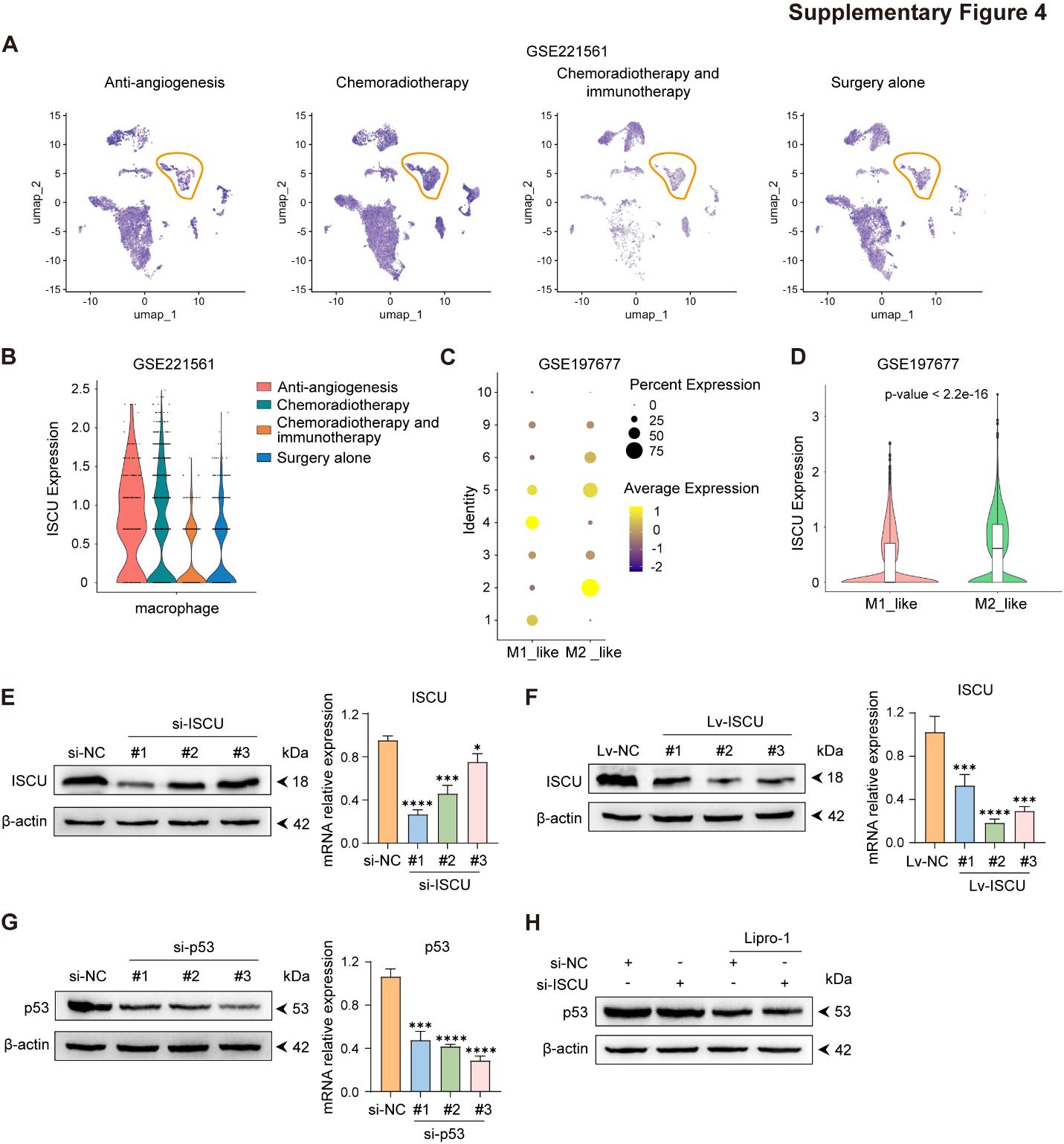


**Fig. S4.**

**A** UMAP plots showing macrophage clusters from the GSE221561 dataset under different treatment conditions. The orange outline highlights macrophage clusters. **B** Violin plot of ISCU expression in macrophages under the four treatment conditions from the GSE221561 dataset. **C** Dot plot showing M1-like and M2-like signature scores among different macrophage clusters from the GSE197677 dataset. **D** Violin plot comparing ISCU expression between M1-like and M2-like macrophages in the GSE197677 dataset. **E** Western blot and qRT-PCR analysis of ISCU protein and mRNA levels in macrophages transfected with si-NC (negative control) or si-ISCU (#1, #2, #3). **F** Western blot and qRT-PCR analysis of ISCU protein and mRNA levels in macrophages transfected with lentivirus (Lv), including lv-NC or Lv-ISCU (#1, #2, #3). **G** Western blot and qRT-PCR analysis of p53 protein and mRNA levels in macrophages transfected with si-NC or si-p53 (#1, #2, #3). **H** Western blot analysis showing p53 expression in macrophages transfected with indicated si-RNAs with or without Liproxstatin-1 treatment. Data are presented as mean ± standard error (n = 3 independent experiments); ns, not significant; ***P < 0.001, ****P < 0.0001 as determined by one-way ANOVA with Tukey’s post-hoc test for multiple comparisons.


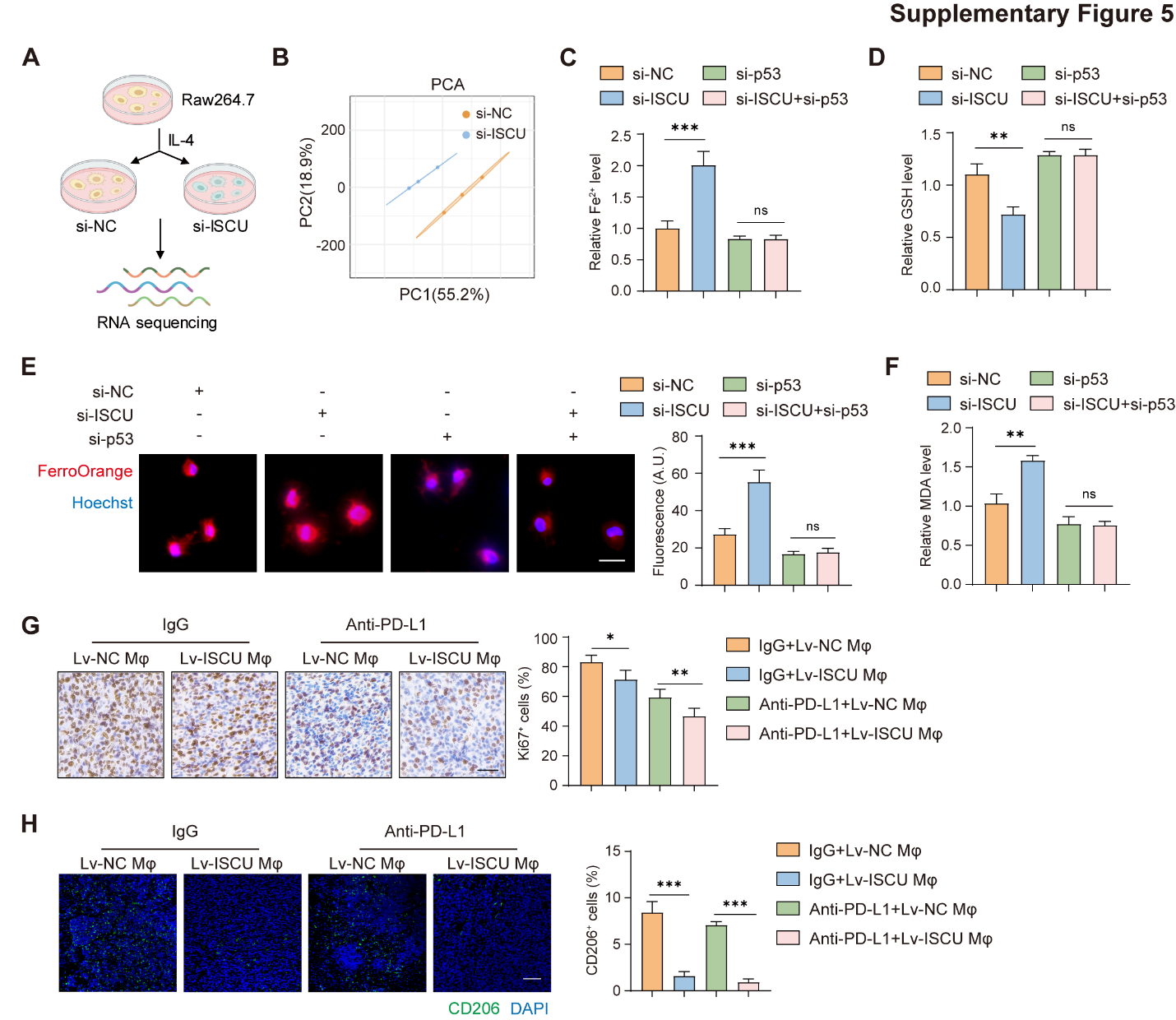
Fig. S5.

**A** Schematic representation outlining the M2 polarization of RAW264.7 macrophages prepared for RNA sequencing. **B** Principal component analysis (PCA) of RNA sequencing data showing distinct clustering of si-NC and si-ISCU groups. **C** Relative Fe²⁺ levels in macrophages transfected with the indicated si-RNAs under M2 polarization conditions. **D** Relative GSH levels in macrophages transfected with the indicated siRNAs under M2 polarization conditions. **E** Representative images of FerroOrange staining (red) and Hoechst staining (blue) in macrophages transfected with the indicated siRNAs under M2 polarization conditions. Quantification of FerroOrange fluorescence intensity is shown on the right. Scale bar: 20 μm. **F** Relative MDA levels in macrophages transfected with the indicated siRNAs under M2 polarization conditions. **G** Immunohistochemical staining of Ki67⁺ cells in tumor tissues from mice under the indicated treatments. Quantification of Ki67⁺ cells is shown on the right. Scale bar: 50 μm. **H** Immunofluorescence staining of CD206⁺ cells (green) and nuclei (DAPI, blue) in tumor tissues from mice under the indicated treatments. Quantification of CD206⁺ cells is shown on the right. Scale bar: 100 μm. Data are presented as mean ± standard error (n = 3 independent experiments); ns, not significant; *P<0.05, **P<0.01, ***P < 0.001, as determined by one-way ANOVA with Tukey’s post-hoc test for multiple comparisons.

Table S1

| **NO.** | **Adult or NOT** | **Immunotherapy** | **chemotherapy** | **Response** | **Pathological evaluation** | **Pathology:**  **prior treatment** | **Pathology:**  **post treatment** |
| --- | --- | --- | --- | --- | --- | --- | --- |
| P972293 | Adult | / | / | / | / | cT2N0M0 | pT1bN0M0 |
| P953374 | Adult | Sintilimab | Docetaxel+  Lobaplatin | PR | pCR | cT3N1M0 | ypTisN1M0 |
| P969124 | Adult | Tislelizumab | TAX+  Paraplatin | PR | PR | cT4N1M0 | ypT2N1M0 |
| P973651 | Adult | Pembrolizumab | TAX+  Paraplatin | SD | MPR | cT3N3M0 | pT3N1M0 |
| P969570 | Adult | Camrelizumab | TAX+  Paraplatin | SD | MPR | cT3N2M0 | ypTisN1M0 |

Table S1 showed the clinical information of five patients, including their detailed treatment regimen and disease staging.

Table. S2.

|  | Forward primer (5’-3’) | Reverse primer (5’-3’) |
| --- | --- | --- |
| *Arg1* | CTCCAAGCCAAAGTCCTTAGAG | AGGAGCTGTCATTAGGGACATC |
| *Fizz1* | CGTGGAGAATAAGGTCAAGGAACT | CACTAGTGCAAGAGAGAGTCTTCGTT |
| *Mrc1* | CTCTGTTCAGCTATTGGACGC | CGGAATTTCTGGGATTCAGCTTC |
| *ISCU* | TCATTATGAAAACCCTCGGAACG | TTTGAATCTGGCGTCCACAAT |
| *Trp53* | CTCTCCCCCGCAAAAGAAAAA | CGGAACATCTCGAAGCGTTTA |
| *TNF-α* | ATGTGGACCCCTCCTGATAGT | GCCCAGTGATTTCAGCAAAGG |
| *iNOS* | TCCTCACTGGGACAGCACAGAATG | GTGTCATGCAAAATCTCTCCACTGCC |
| *IL-6* | GTCCGGAGAGGAGACTTCAC | CTGCAAGTGCATCATCGTTGT |
| *β-actin* | GGCTGTATTCCCCTCCATCG | CCAGTTGGTAACAATGCCATGT |

Table S2 showed the primers used for quantitative real-time PCR in this study.
