# Supplementary material for: ISCU-p53 axis orchestrates macrophage polarization to dictate immunotherapy response in esophageal squamous cell carcinoma
Source: Cell Death Dis. 2025 Jun 20;16(1):462. doi: 10.1038/s41419-025-07787-7 (PMC12181301; doi:10.1038/s41419-025-07787-7)

Figure 4H

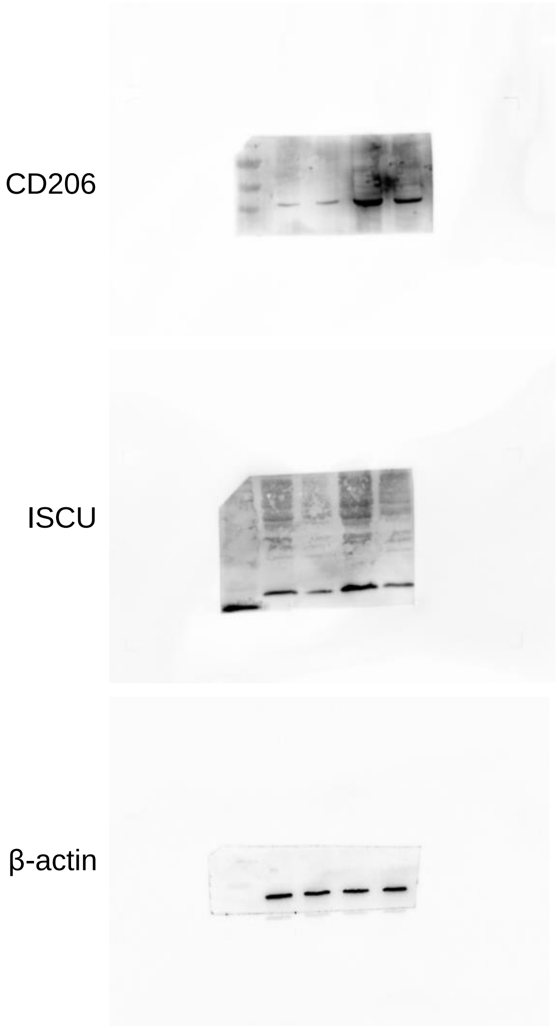

Figure 5D

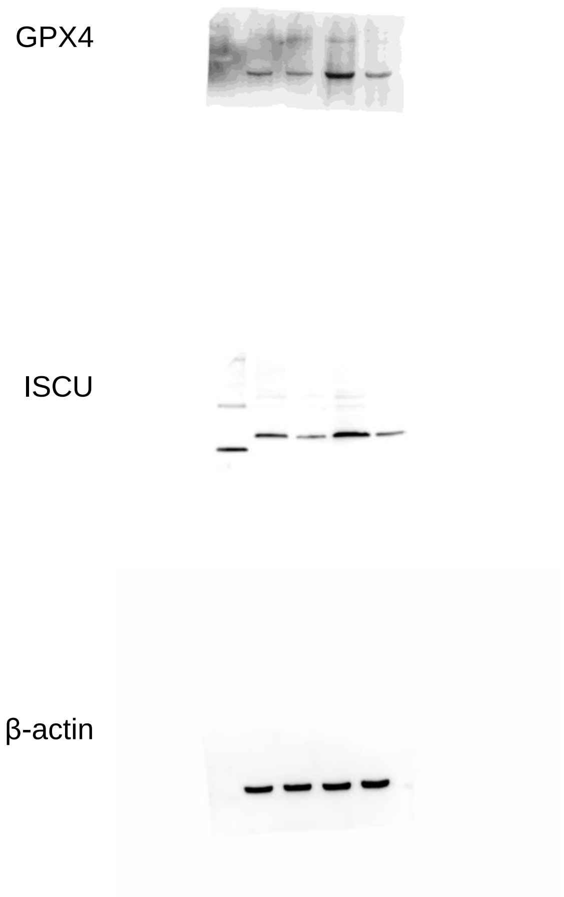

Figure 5K

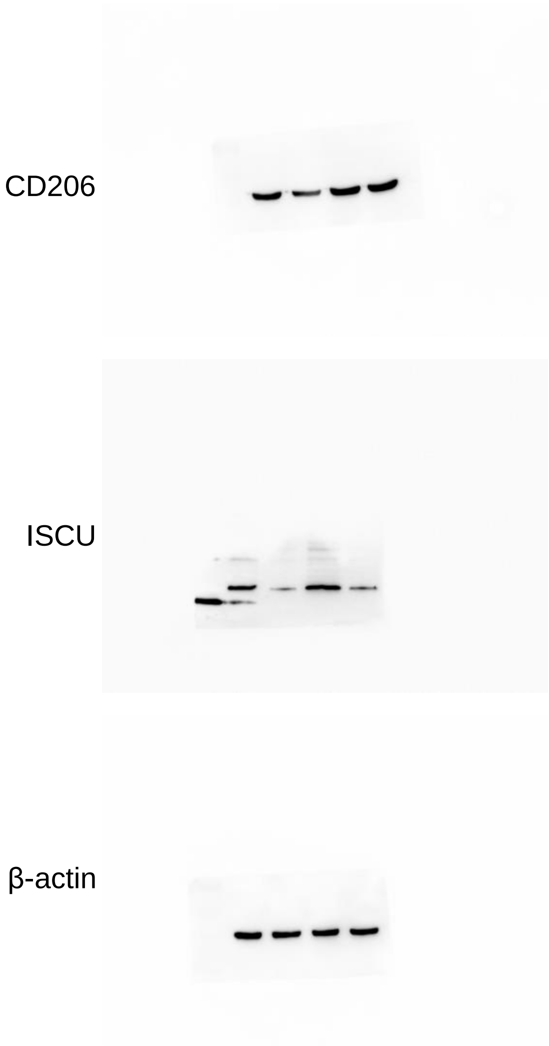

Figure 6C

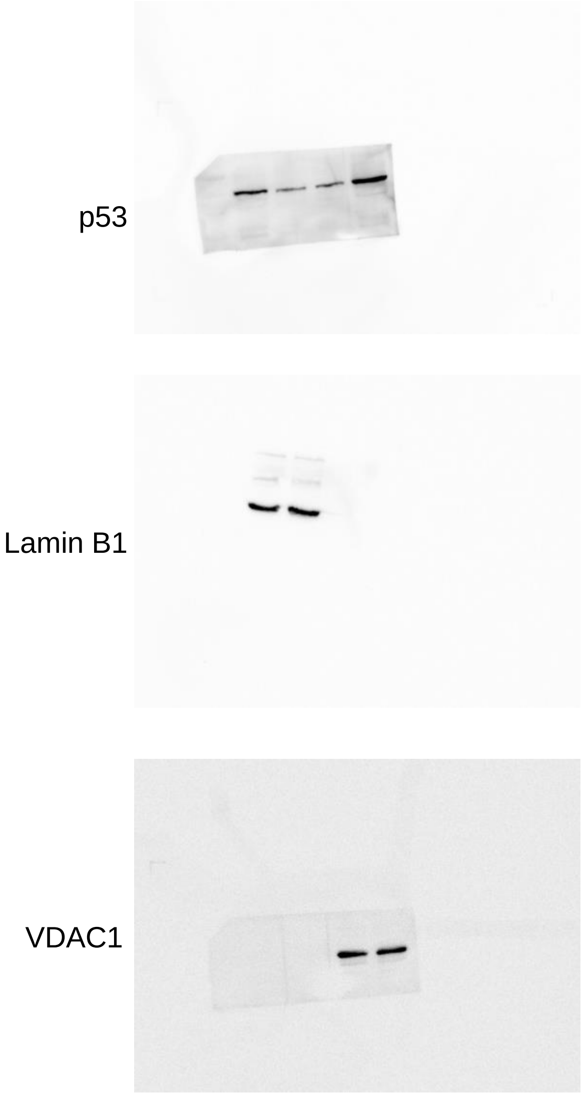

Figure 6D

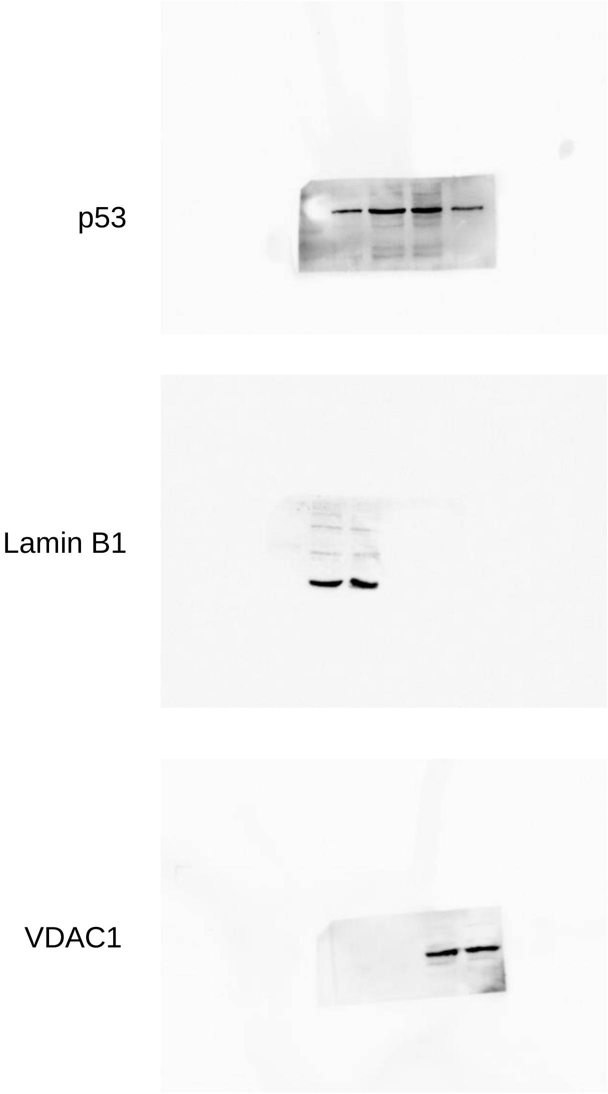

Figure 6E

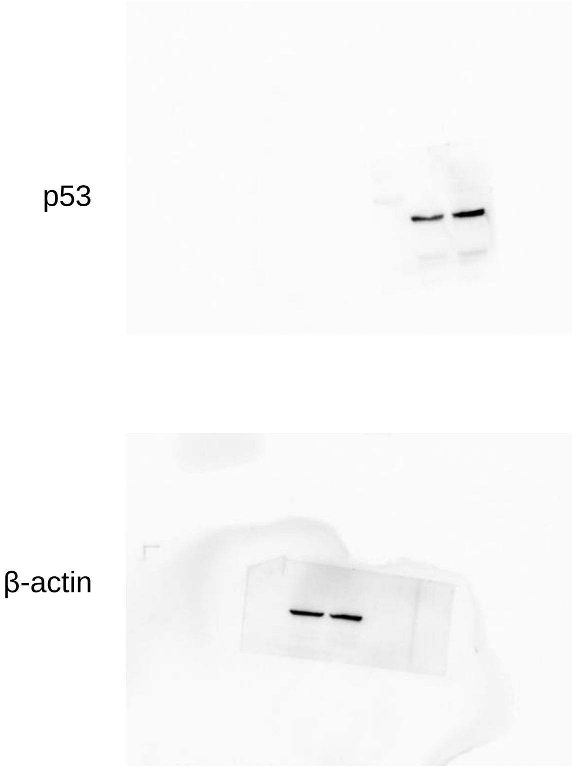

Figure 6F

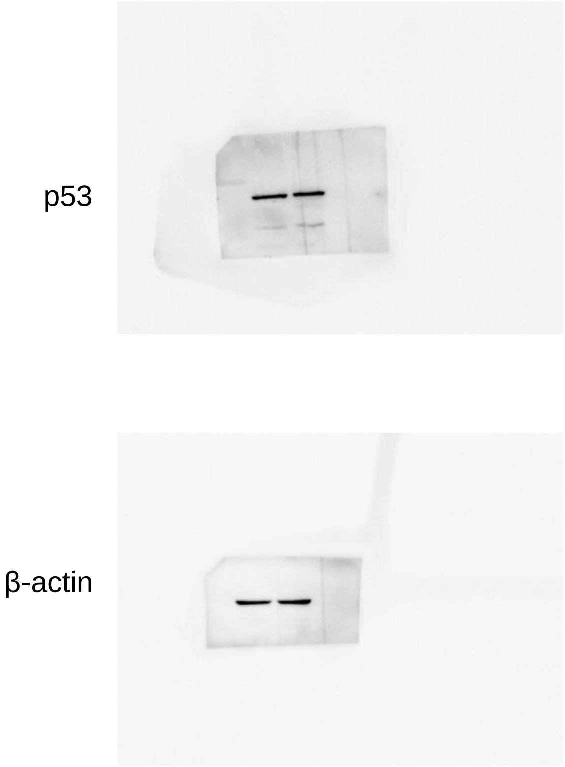

Figure 6G left panel

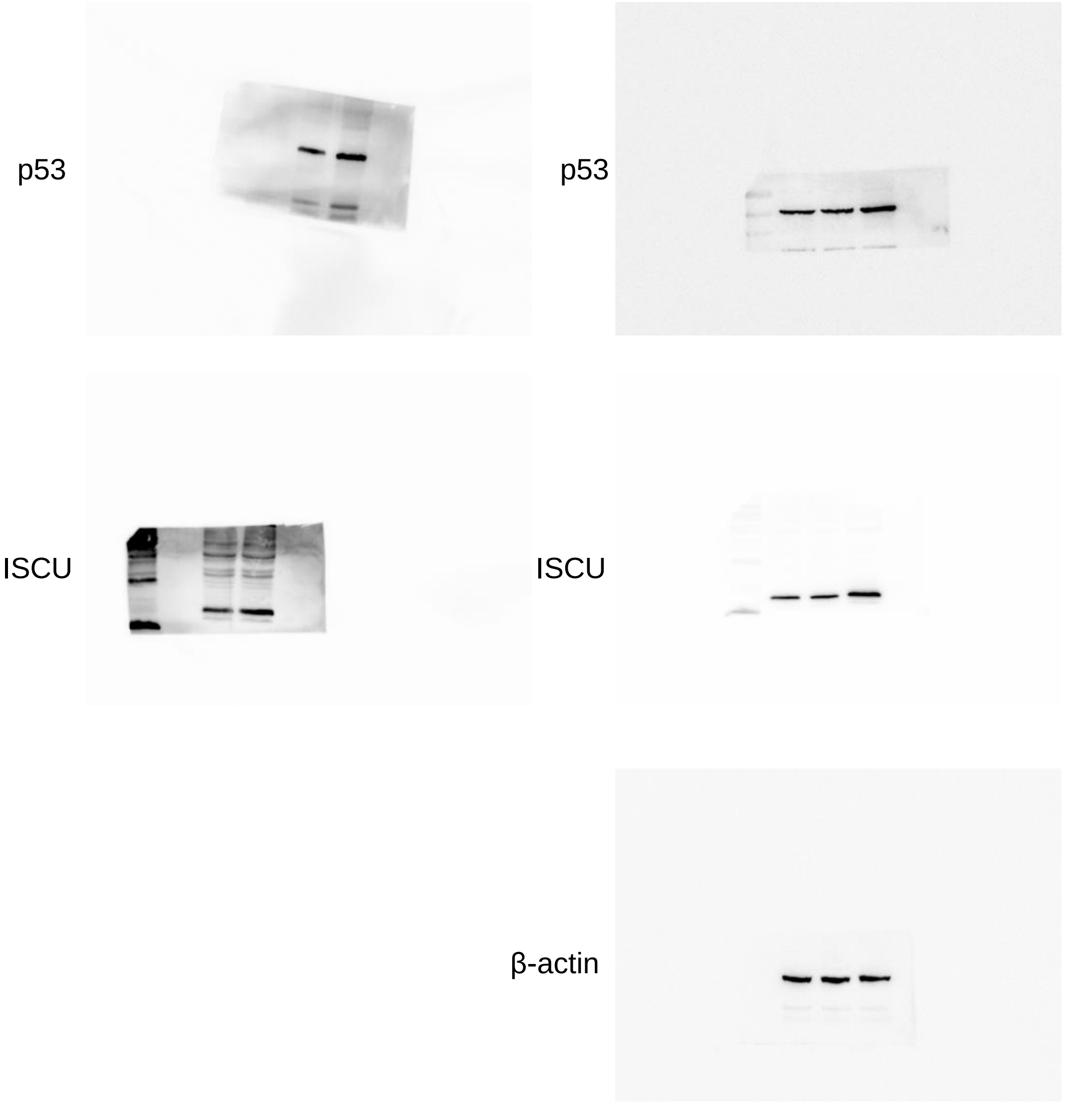

Figure 6G right panel

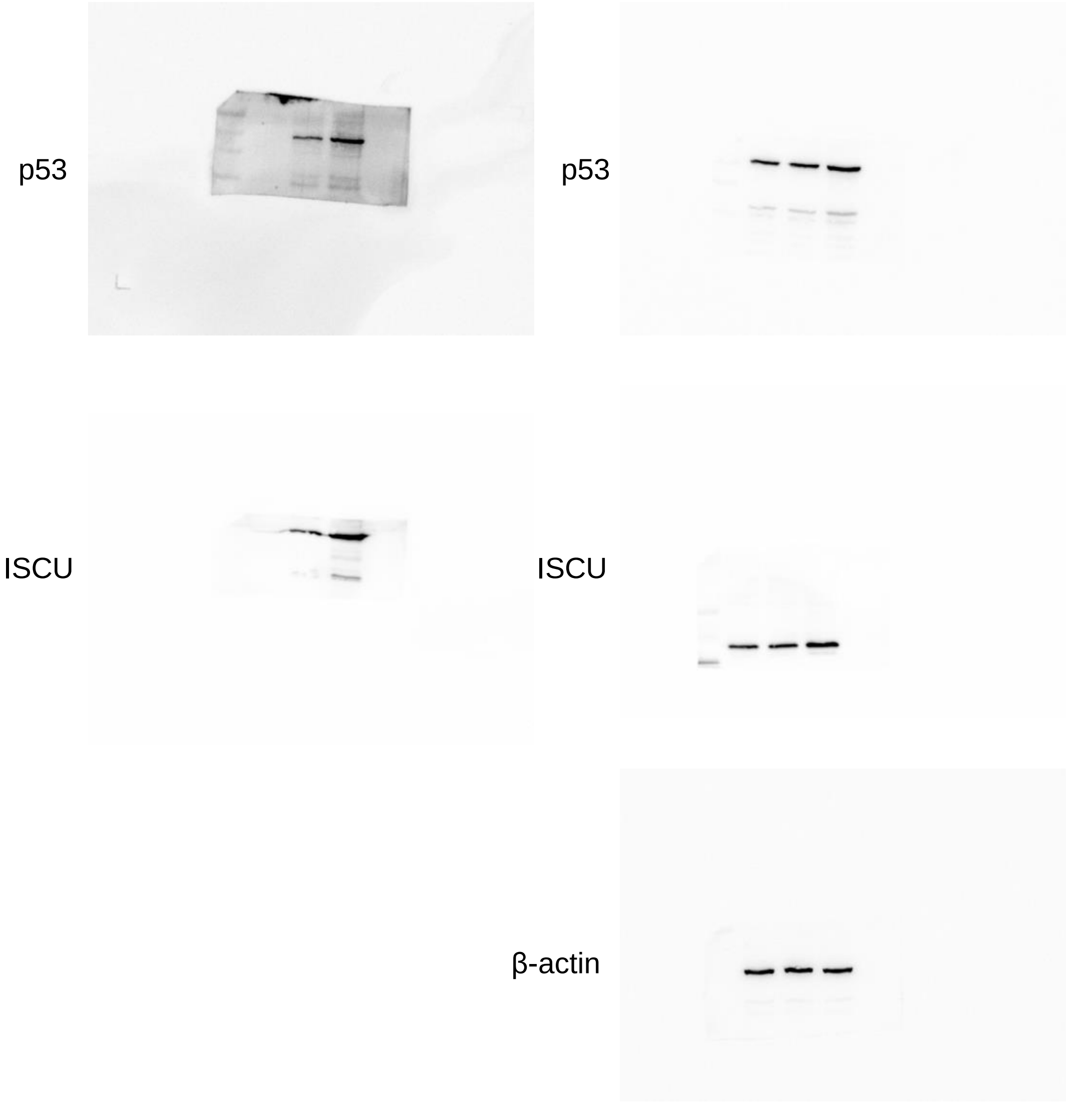

Figure 6H left panel

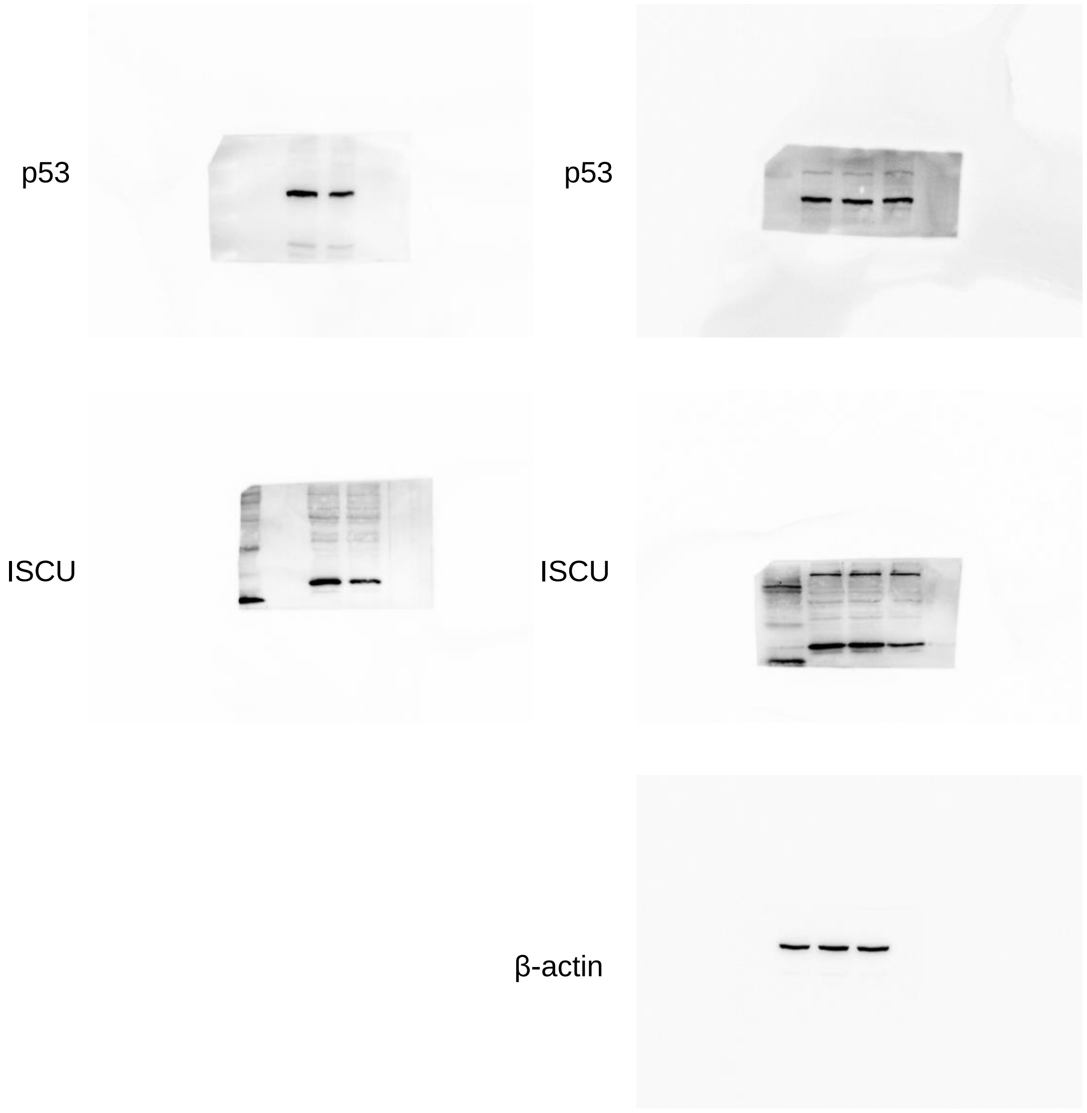

Figure 6H right panel

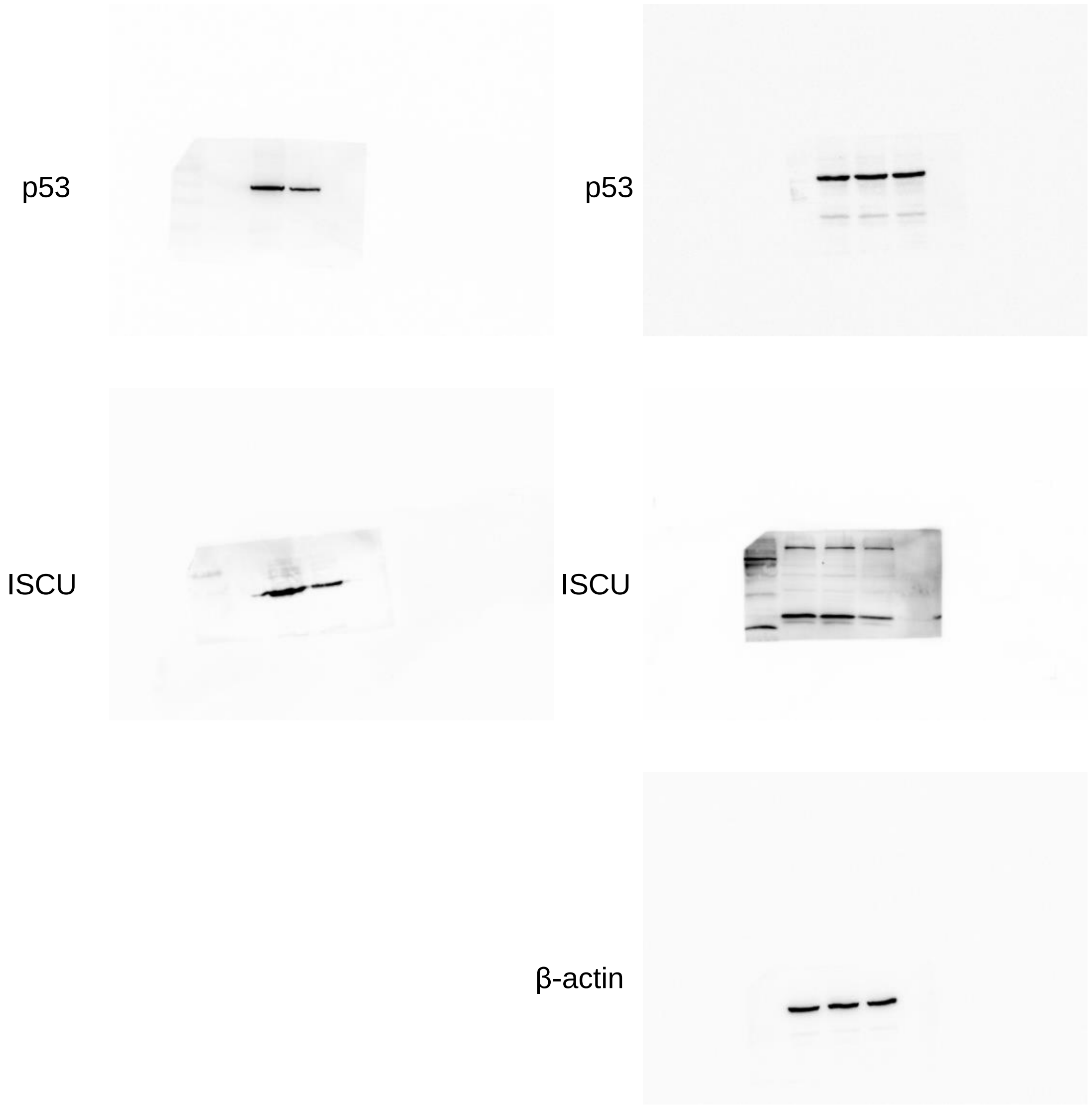

Figure 7C

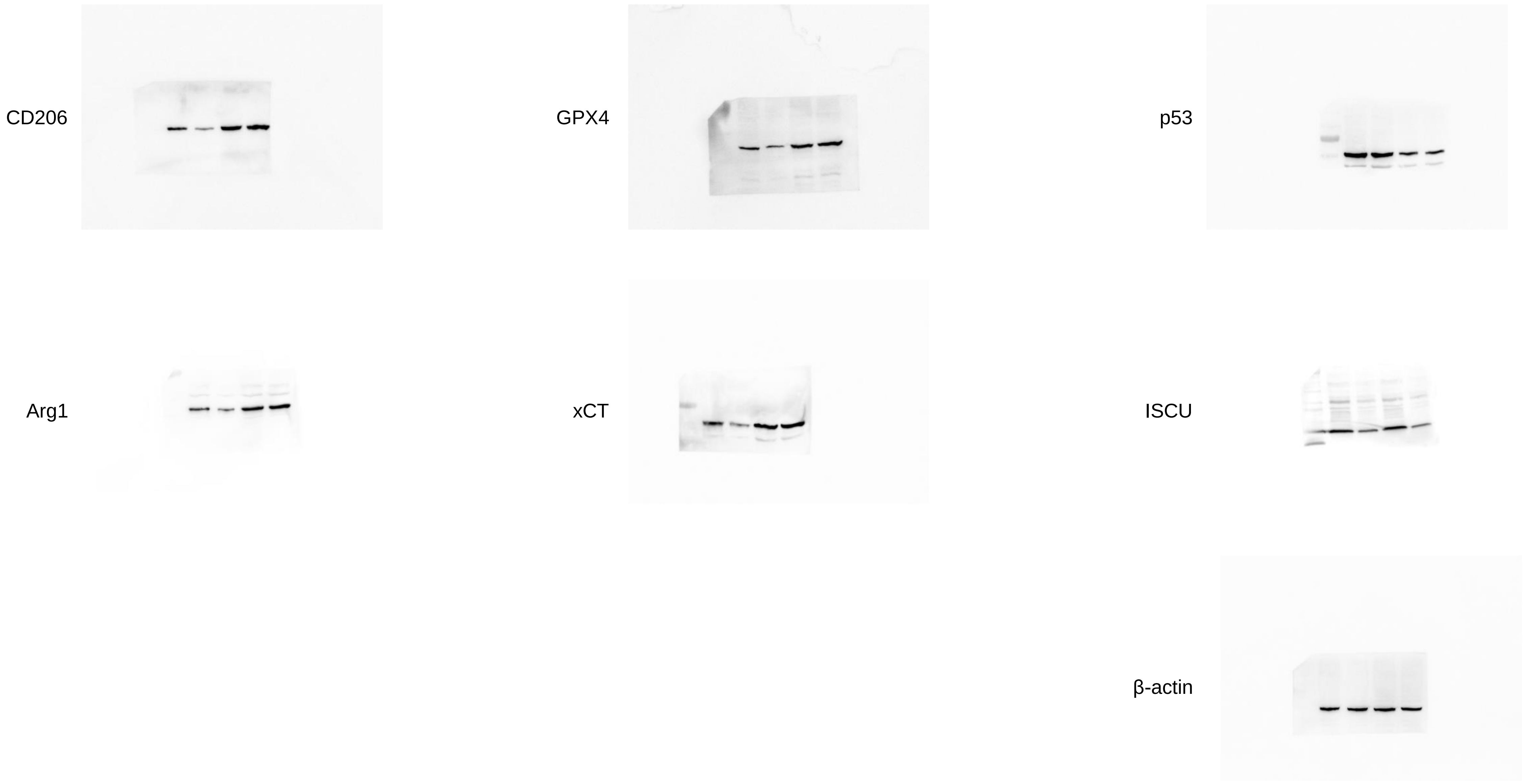

Figure 8N

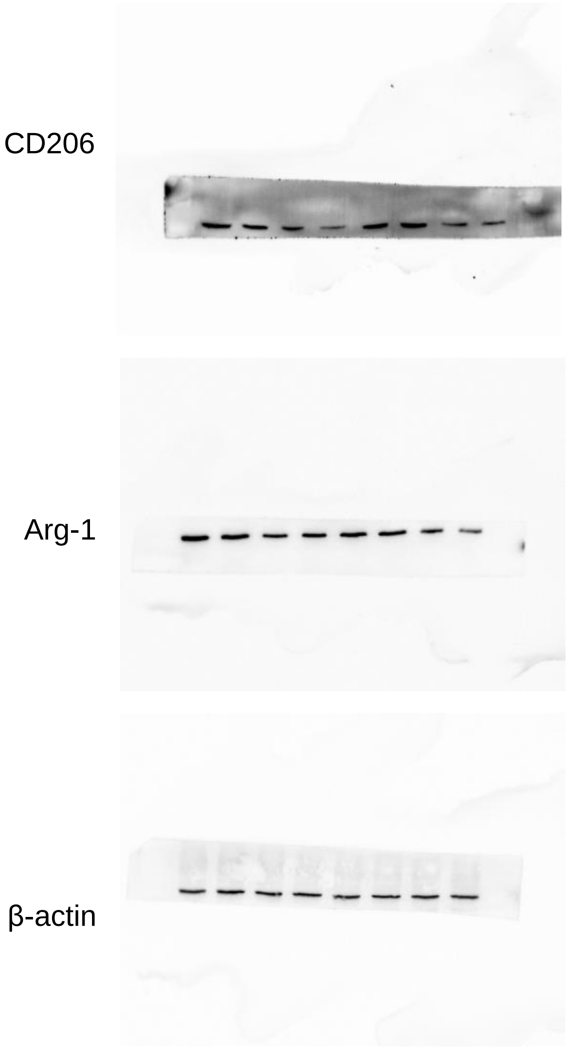

Supplementary Figure 4E

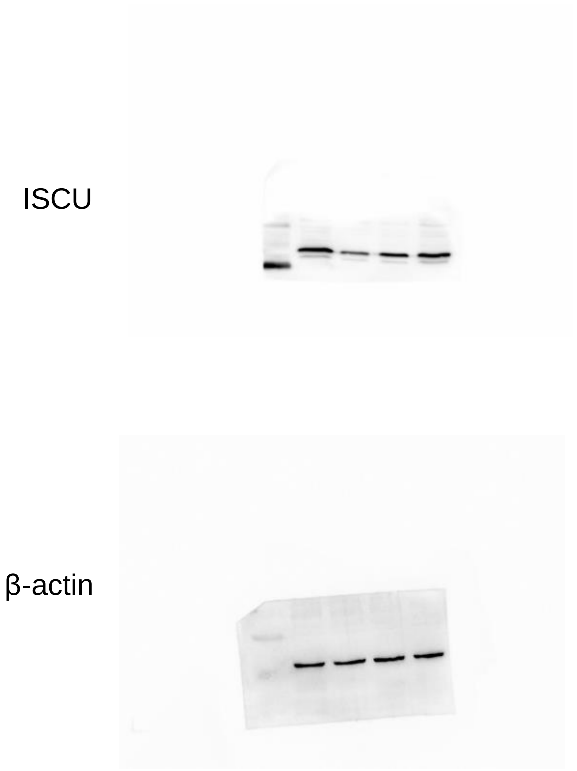

Supplementary Figure 4F

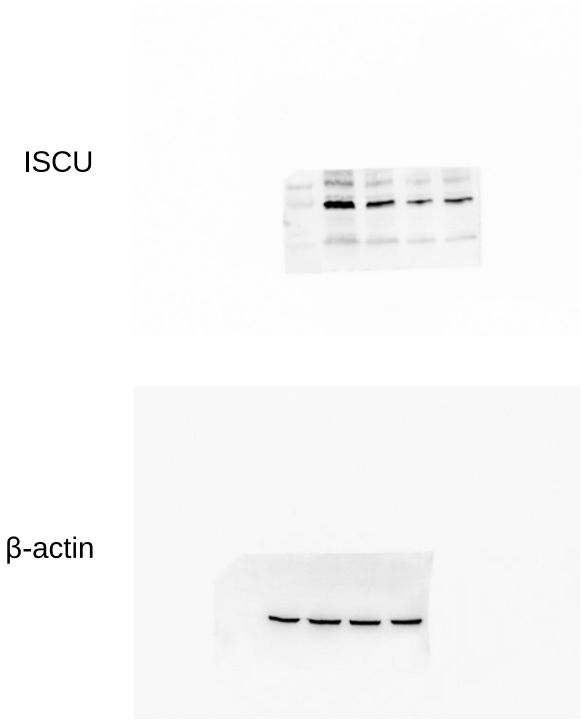

Supplementary Figure 4G

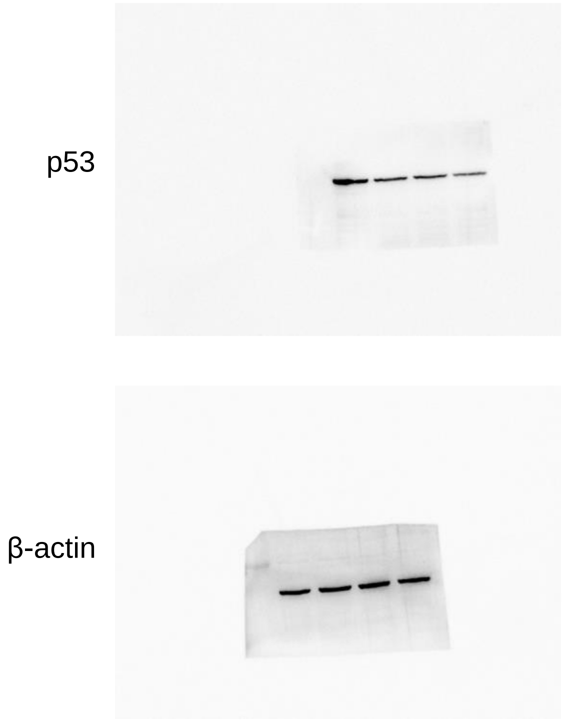

Supplementary Figure 4H

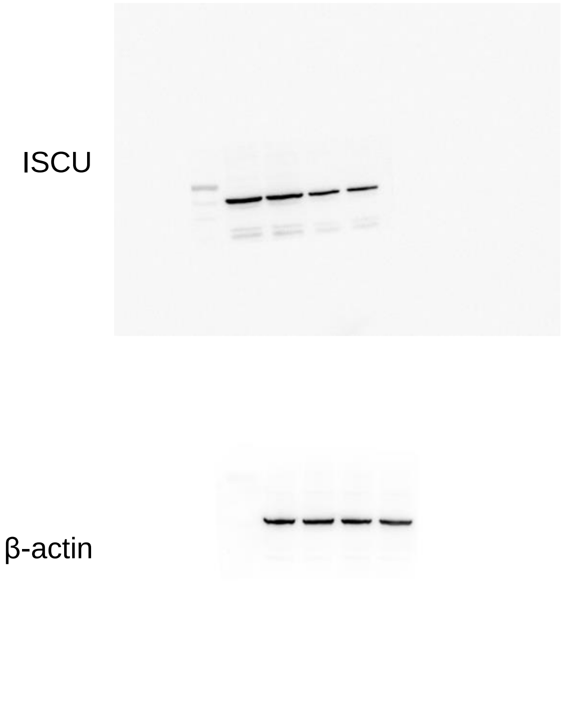

Supplement: Supplementary file 2 — Western blots [file 41419_2025_7787_MOESM2_ESM.pdf]
